# Good practice characteristics of diet and physical activity interventions and policies: an umbrella review

**DOI:** 10.1186/s12889-015-1354-9

**Published:** 2015-01-21

**Authors:** Karolina Horodyska, Aleksandra Luszczynska, Matthijs van den Berg, Marieke Hendriksen, Gun Roos, Ilse De Bourdeaudhuij, Johannes Brug

**Affiliations:** 1 grid.433893.60000000121840541Department of Psychology, University of Social Sciences and Humanities, 30b Ostrowskiego St, 53238 Wroclaw, Poland; 2 Trauma, Health, & Hazards Center, University of Colorado, 1861 Austin Bluffs Pkwy, Colorado Springs, CO 80933-7150 USA; 3 grid.31147.300000000122080118National Institute for Public Health and the Environment, Antonie van Leeuwenhoeklaan 9, 3721 Bilthoven, the Netherlands; 4 SIFO – National Institute for Consumer Research, Sandakerveien 24 C, Building B Oslo, P.O. BOX 4682, Nydalen, N-0405, Oslo Norway; 5 grid.5342.00000000120697798Department of Movement and Sport Sciences, Ghent University, Watersportlaan 2, 9000 Ghent, Belgium; 6 grid.16872.3a000000040435165XVU University Medical Center, Amsterdam, Van der Boechorststraat 7, 1081 BT Amsterdam, the Netherlands

**Keywords:** Physical activity, Sedentary behavior, Diet, Good practice, Intervention, Policy, Systematic review

## Abstract

**Background:**

This umbrella review aimed at eliciting good practice characteristics of interventions and policies aiming at healthy diet, increasing physical activity, and lowering sedentary behaviors. Applying the World Health Organization’s framework, we sought for 3 types of characteristics, reflecting: (1) main intervention/policy characteristics, referring to the design, targets, and participants, (2) monitoring and evaluation processes, and (3) implementation issues. This investigation was undertaken by the DEDPIAC Knowledge Hub (the Knowledge Hub on the DEterminants of DIet and Physical ACtivity), which is an action of the European Union’s joint programming initiative.

**Methods:**

A systematic review of reviews and stakeholder documents was conducted. Data from 7 databases was analyzed (99 documents met inclusion criteria). Additionally, resources of 7 major stakeholders (e.g., World Health Organization) were systematically searched (10 documents met inclusion criteria). Overall, the review yielded 74 systematic reviews, 16 position review papers, and 19 stakeholders’ documents. Across characteristics, 25% were supported by ≥ 4 systematic reviews. Further, 25% characteristics were supported by ≥ 3 stakeholders’ documents. If identified characteristics were included in at least 4 systematic reviews or at least 3 stakeholders’ documents, these good practice characteristics were classified as relevant.

**Results:**

We derived a list of 149 potential good practice characteristics, of which 53 were classified as relevant. The main characteristics of intervention/policy (*n* = 18) fell into 6 categories: the use of theory, participants, target behavior, content development/management, multidimensionality, practitioners/settings. Monitoring and evaluation characteristics (*n* = 18) were grouped into 6 categories: costs/funding, outcomes, evaluation of effects, time/effect size, reach, the evaluation of participation and generalizability, active components/underlying processes. Implementation characteristics (*n* = 17) were grouped into eight categories: participation processes, training for practitioners, the use/integration of existing resources, feasibility, maintenance/sustainability, implementation partnerships, implementation consistency/adaptation processes, transferability.

**Conclusions:**

The use of the proposed list of 53 good practice characteristics may foster further development of health promotion sciences, as it would allow for identification of success vectors in the domains of main characteristics of interventions/policies, their implementation, evaluation and monitoring processes.

**Electronic supplementary material:**

The online version of this article (doi:10.1186/s12889-015-1354-9) contains supplementary material, which is available to authorized users.

## Background

According to the World Health Organization (WHO) low levels of physical activity constitute the fourth leading risk factor for death worldwide and form a key risk factor for non-communicable diseases such as cancer, diabetes, and cardiovascular diseases [1]. Inadequate diet is related to increased likelihood of developing obesity, increased susceptibility to diseases such as diabetes and cardiovascular diseases, reduced immunity, and reduced productivity [1]. Therefore, as suggested by WHO [1] interventions and policies which focus on diet, physical activity, or sedentary behavior are in the main focus of various science disciplines, health organizations, practitioners, and policy makers. Unhealthy diet and physical inactivity are considered among leading causes of the same set the major non-communicable diseases [1], therefore interventions and policies targeting either one of these behaviors or both of them are considered complementary, serving the same overall goals, and they are guided by the same principles for action [1].

Policies constitute of a purposive course of actions to stimulate a healthy diet, physical activity, or to discourage sedentary behavior (defined as the amount of time per day spent sitting, in non-active activities such as watching TV, working at a computer, reading etc.). Policies are formulated in a specific political process; they are adopted, implemented, and enforced by regional, national or international public agencies [2]. In contrast, interventions are actions not yet endorsed, enabled or executed by governments or other public agencies. Interventions may address individuals’ skills, individuals’ beliefs, and contexts such as social systems, physical or build environment, or they may focus on practicing recommended behavior during the intervention sessions. Multilevel and multicomponent interventions may combine these actions and aim at changes at individual, social, and physical environmental levels. Such interventions and policies may have the greatest potential to be effective and thus they may be appealing to practitioners and funding bodies [3]. However, high complexity of interventions and policies hinders identification of the factors responsible for their success.

### Good practice characteristics of interventions and polices

Although the number of studies on developing and testing the effects of interventions and policies is growing rapidly, practitioners, policy-makers and researchers indicate difficulties in eliciting factors responsible for a ‘success’ of interventions or policies [4]. ‘Successful’ interventions or policies may be defined as actions that result in significant and sustainable behavior changes and translate behavior change research into real-word settings [5]. Such successful interventions and policies may be characterized by a number of good practice characteristics. These characteristics may include the content of behavior change techniques [6] or aspects of delivery of these techniques [7]. Another line of research stresses that besides the content of an intervention or policy, other characteristics determining ‘successful’ promotion of healthy behaviors may refer to implementation strategies, settings, or integration with local practice [8].

Several conceptual frameworks propose the list of good practice characteristics, defined as characteristics of successful interventions and policies [9,10]. Those lists vary in terms of the range of included characteristics, and in terms of the breadth of the scope. For example, they may focus on specific populations (e.g., children only) [10] or on aspects of implementation (e.g., fidelity to the protocols, consistent delivery) [9]. Although empirical evidence and theoretical developments are accumulating, we found no list of generic characteristics (e.g., non-specific in terms of population, addressing both policies and interventions), which would account for content, evaluation, and implementation aspects.

To identify a good practice characteristic that is typical of ‘successful’ interventions or policies one needs to establish a list of ‘candidate characteristics’ , which have a *potential* to determine a success. In case of some characteristics, the evidence accumulated in systematic reviews suggests that the presence (or absence) of a characteristic is linked to effects of interventions/policies on diet, physical activity, and sedentary behavior. The development of an evidence-based list of candidate characteristics may serve several aims, namely (1) it may inform the development of new interventions and policies by indicating areas, which should be considered when planning for new interventions/policies; (2) the list may be used as an extended protocol for reporting on interventions and policies; (3) it may promote collection of extended data on characteristics of interventions/policies; collection of these data would enable identifying the essential criteria of successful health promotion.

### Reporting and categorizing characteristics of interventions and policies

A progress towards the development and synthesis of interventions and policies is hindered by a lack of widely approved standards of reporting interventions and policies [4]. Existing checklists and protocols for reporting interventions and policies provide some details, but their depth and breadth are limited. The guidelines for reporting are relatively vague, and thus there is not enough information for thorough replications of complex interventions or policies. For example, CONSORT guidelines [11] require reporting “precise details” of the interventions/policies and indicating “how and when they were actually administered”, therefore a very limited description of procedures may fit these standards. To further aid reporting guidelines, Davidson and colleagues [12] suggested that reports of behavior change actions should include details of: (1) the content, (2) characteristics of those delivering the intervention, (3) characteristics of the recipients, (4) settings (e.g., worksite), (5) the mode of delivery, (6) intensity of actions, (7) their duration, and (8) adherence to delivery protocols. Unfortunately, this proposal uses broad constructs; for example it does not specify the type of characteristics of recipients which may be relevant (e.g., minority status, age, beliefs). In sum, these proposals use broad and unspecific characteristics or leave out many characteristics (e.g., implementation, resources [9]) which may determine a success of interventions or polices.

There are several theoretical frameworks which may inform the organization of good practice characteristic. These frameworks tackle the complexity of characteristics, but they usually emphasize either the aspect of content or implementation, or evaluation processes. For example, the Behavior Change Wheel [13] focuses on the content of the interventions or policies, whereas other approaches such as RE-AIM model [9] focus on implementation processes. Another approach to organizing good practice characteristics was recently proposed by WHO [14]. This framework aims at eliciting and classifying good practice characteristics in actions targeting healthy diet and physically active lifestyle. Good practice characteristics were grouped in 3 domains: (1) main intervention/policy characteristics (including the general design, content, main objectives, planned activities, target groups, and stakeholders), (2) monitoring and evaluation (including outcomes, measurement, and process evaluation aspects), and (3) implementation (including performance of implementation, program management, and participation processes). This broad framework was validated in consultations with stakeholders and pilot tests conducted among large-scale program developers [14].

### Aims

As the part of the investigation undertaken by the DEDPIAC project (the Knowledge Hub on the DEterminants of DIet and Physical Activity, which is the first Research Action of the European Union’s Joint Programming Initiative on healthy diet for healthy life) [15], the present study aimed at identifying characteristics of interventions and policies promoting healthy diet, physical activity, and a reduction of sedentary behavior. To achieve this target, we performed an umbrella review (i.e., a systematic review of existing reviews) which integrated empirical evidence from existing systematic reviews, position review papers, and stakeholders’ documents. The questions and methods were developed and approved using the rapid review approach [16]. We investigated the presence of attributes of good or recommended practices in policies and interventions targeting the general population, children, and adults. Applying the best practice framework [14], we sought for (1) main intervention/policy characteristics, (2) monitoring and evaluation characteristics, and (3) implementation characteristics.

## Methods

### Materials and general procedures

We conducted the umbrella review to identify systematic reviews and stakeholders’ documents. In general, systematic reviews and meta-analyses collate empirical evidence that fits pre-specified eligibility criteria, by using explicit, replicable, systematic search, extraction, and evaluation methods that are selected to minimize biases [17]. Umbrella reviews represent a way of synthesizing the evidence accumulated in systematic reviews and making them suitable for a more general audience of healthcare practitioners [18-20]. Although typical umbrella reviews focus on analyzing materials obtained from systematic reviews [17,18], the aim of this study required integrating the evidence presented in reviews (both systematic and non-systematic position reviews) with practice recommendations issued by major stakeholders.

In order to elicit the good practice characteristics 3 types of documents were analyzed. First, we searched for systematic reviews analyzing characteristics of policies/interventions, and forming recommendations about these characteristics. Second, we searched for position papers that offered a comprehensive review of research evidence supporting good practice characteristics, but did not apply methods of systematic reviews. Finally, we searched for peer-reviewed and non-peer-reviewed documents, issued by major national and international stakeholders. We investigated documents aiming at eliciting evidence-based good practice criteria or providing practice recommendations for interventions/policies targeting healthy diet, physical activity, or sedentary behaviors.

### Peer-reviewed documents: search strategy, inclusion, and exclusion criteria

The search was conducted in Medline, Cochrane Database of Systematic Reviews, PsycINFO, PsychArticles, Health Source: Nursing/Academic Edition, Academic Premier, and ScienceDirect databases. Documents published between the inception of databases and February 2014 were included. Combinations of 4 groups of keywords were applied, referring to: (1) practice characteristics (“good practic*” or “best practic*” or “recommended practic*” or “recommended strateg*”), (2) the type of action (intervention or polic*), (3) the design (“systematic review” or review or meta-analys*), (4) diet, physical activity, or sedentary behavior-related outcomes (“physical activity” or active or exercise or sedentary or diet or nutrition or fat or snack or fruit or vegetable or fiber or fibre or soda or meal or food or “energy intake” or calorie* or obes*).

**Figure 1:**
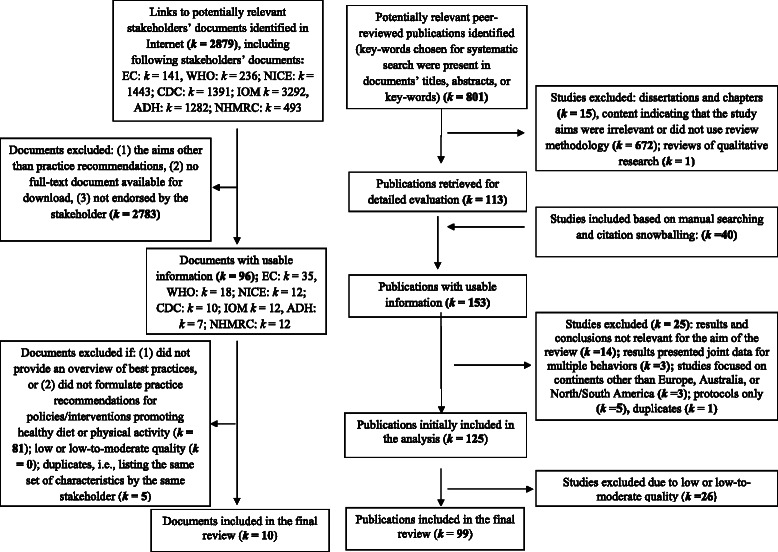
**The flow chart: Selection processes for stakeholders’ documents (left panel) and reviews (right panel).**

Figure 1 (right panel) presents the stages of the data selection process. The preliminary search yielded 1926 entries, which used a combination of keywords from all 4 categories in either title, or the abstract, or keywords. Identified abstracts were then screened by 2 researchers (KH and AL), and 801 potentially relevant studies were identified.

The researchers (KH and AL) then selected publications that appeared in peer-reviewed English language journals. The following documents were excluded: (a) dissertations, protocols, conference materials, and book chapters; (b) reviews which indicated a need for testing good practice characteristics, but did not investigate such characteristics in the Results sections; (c) documents analyzing interventions or policies focusing on other main outcomes than physical activity, sedentary behaviors or diet. For example, interventions could target prevention or treatment of osteoporosis; such interventions could account for physical activity or diet (listed among multiple behaviors requiring change) but the content, evaluation, and implementation characteristics of such interventions were specific for their respective main outcomes (e.g., focusing on characteristics of prior treatment, current medication, health maintenance organizations); (d) publications aiming at eliciting practice characteristics in multi-behavior interventions/polices, which did not distinguish characteristics specific for either dietary or physical activity or sedentary behaviors; (e) documents which were reviewing guidelines for diet/physical activity/sedentary behaviors, but did not indicate characteristics of interventions/policies; (f) publications which discussed only one example of a policy or intervention.

In case of *systematic reviews* we included systematic reviews of quantitative studies (criteria for systematic reviews: clearly defined study aims, search strategy, inclusion and exclusion criteria, design of original studies, a suitable synthesis given the heterogeneity of findings [17]). In case of *non-systematic position reviews*, papers focusing on eliciting evidence-based good practices were included. Regarding *peer-reviewed stakeholder’s documents*, we included papers indicating a major professional organization (e.g., American Dietetic Association) among the authors or in the title. If several position review papers were prepared by the same authors and dealt with the same original trials, we included the most recent document, and sought for other (distinct) characteristics in earlier documents. Further, manual searches of the reference lists were conducted.

*Quality assessment* of each systematic review was conducted using the Methodological Quality Checklist (MQC) [18]. It is a 7-item scale with total scores ranging from 0 to 7. MQC evaluates strategies applied in original reviews and accounts for 7 quality criteria: (1) well-defined study participants, intervention, and outcomes; (2) search strategy is defined, combining several databases and other strategies of reference checking; (3) transparent inclusion and exclusion criteria; (4) designs of original studies and the number of studies are clarified; (5) quality assessment of original studies is included; (6) methods of data synthesis is specified and it accounts for data heterogeneity; and (7) at least 2 researchers are involved at each stage of review process. Two researchers (KH and AL) rated all systematic reviews independently. Previous umbrella reviews using MQC applied the cutoff of 4 as representing moderate or high quality [19,20] and included research which scored ≥ 4 in MQC. Therefore, only systematic reviews scoring ≥ 4, were included into the final analyses.

Because there are no widely used measures of quality evaluation of stakeholders’ documents or non-systematic reviews, we have developed a tool serving this purpose (the Methodological Quality Checklist for Stakeholder Documents and Position Papers, MQC-SP; see Additional file 1). It was based on criteria applied in MQC [18], with a 6-item scale and total scores ranging from 0 to 6. Two researchers (KH and AL) independently rated all peer-reviewed stakeholders’ documents and position review papers. Only papers scoring ≥ 4, representing moderate or high quality, were included into analyses.

For all 3 types of analyzed documents the concordance of quality evaluations was high. The values of kappa coefficient were ranging from .89 (*p* < .01) to 1.00 (*p* < .001).

Overall, 99 peer reviewed papers met all inclusion and exclusion criteria. They included 74 systematic reviews, 16 position papers based on a review of empirical evidence, and 9 stakeholders’ documents, published in peer-reviewed journals.

### Stakeholders’ documents (other than peer-reviewed): search strategy, inclusion criteria, exclusion criteria, and quality evaluation

Besides identifying the stakeholders’ documents that were published in peer-reviewed journals, we searched for documents published directly by the stakeholders. To obtain *major stakeholders’ documents* aiming at good practice recommendations, a group of 5 experts used the consensus method [17] to select the stakeholders. The inclusion criteria were: (1) the international or national stakeholder using English language to issue the documents (i.e., developing documents available to researchers, policy makers, and practitioners around the world); (2) the stakeholder issues documents that provide evidence-based good practice recommendations for policies and interventions targeting any populations; (3) the stakeholder develops documents which refer to diet, physical activity, or sedentary behavior as the main outcome of the interventions/policies. Similar inclusion criteria were used in previous reviews of stakeholders’ documents [21]. The following stakeholders were included: European Commission (EC), National Institute for Health and Clinical Excellence (NICE; United Kingdom), World Health Organization, Regional Office for Europe (WHO), Centers for Disease Control and Prevention (CDC; USA), Institute of Medicine (IOM; USA), Australian Department of Health (ADH), and National Health and Medical Research Council (NHMRC; Australia). All websites of respective stakeholders were searched using the same keywords as in the search of the databases. Using a search engine (Google) further attempts to elicit potential documents were undertaken. The sets of keywords used in the databases search were applied, accompanied by the names of the stakeholder organization (or its acronym). Only documents that were available for downloading were included. The initial search resulted in identifying 8279 links to potentially relevant documents (see Figure 1, left panel).

In the next step, the titles of these potentially relevant documents were screened and the documents with titles that appeared relevant for the present umbrella review were further reviewed. We included documents which aimed at (1) reviewing good practices or (2) providing an overview of characteristics of good practices, or (3) formulating practice recommendations in interventions or policies promoting healthy diet, physical activity, or a reduction of sedentary behavior. Only documents developed (or officially endorsed) by a respective stakeholder were included. Documents were excluded if they presented selected examples of good practice in interventions/policies or if they did not focus on the characteristics of interventions/policies. Moreover, we excluded documents aiming at interventions or policies focusing on other main outcomes than physical activity, sedentary behaviors or diet (e.g., osteoporosis prevention). These steps were conducted by 2 researchers (MH and MvdB).

Next, the quality of the documents was evaluated. The quality criteria were based on MQC criteria [18], with a 6-item scale and total scores ranging from 0 to 6 (see Additional file 1). Two researchers (MH, MvdB) independently rated all stakeholders’ documents. Papers scoring ≥ 4, that is representing high or moderate quality, were included into the further analyses. The concordance of the quality evaluation was very high, with κ = 1.00, *p* < .001.

Overall, we obtained 15 non-peer-reviewed stakeholder documents, meeting all inclusion criteria. However, 5 out of 15 documents presented a major overlap with another document issued by the same stakeholder; such documents were excluded (i.e., the excluded document did not report any additional practices compared to the included document). Thus, 10 documents were used for further analyses.

### Data extraction, coding, and synthesis

To ensure accuracy and consistency of data extraction and coding at least 2 researchers extracted and coded data independently. Any disagreements in the processes of data selection and abstraction were resolved by the consensus method (searching for possible rating errors, followed by a discussion and arbitration by a third researcher) [17].

*Descriptive data* was extracted by one researcher (KH or MH) and then verified by the second researcher (AL). Extracted data included: (1) the descriptive characteristics of the original studies (e.g., participants, target behavior), synthesized in the analyzed reviews and stakeholders’ documents; (2) data necessary for quality evaluation. These descriptive characteristics and quality evaluations are presented in Additional file 1.

Next, the *intervention and policy characteristics* were extracted. Each document was searched for good practice characteristics. In particular, we retrieved the names of characteristics (as documented by authors of original documents) and their operationalization or definition (e.g., an explanation of the function of a characteristic within the context of development, implementation, and evaluation of policies and interventions). In case of systematic reviews these characteristic had to be included in the original analysis (as a significant determinant or as a moderator) as well as into original conclusions of the review. In case of stakeholders’ documents and position review papers, attributes of intervention/policies listed in the respective documents were coded as good practice characteristics if they were indicated as crucial for the development, implementation, and evaluation of any interventions or policies targeting healthy diet and physically active lifestyle.

The good practice characteristics that had an equivalent operationalization but different original names were considered to represent the same construct (e.g., accounting for cultural customs and addressing ethnic minority values). If an original document used a broad name for a characteristic (e.g., costs) we elicited a functional definition applied in the original document (e.g., total costs, cost per participants, cost per a unit of behavior change), and the broader characteristic was divided into separate units, reflecting its functional definition. The findings are presented using definitions as presented by the authors of the original documents (see Additional file 1). Interventions and policies aiming at any type of physical activity (general levels of physical activity or its specific types, such as walking) or a reduction of sedentary behavior were coded as referring to physical activity. Only 4 documents addressed sedentary behaviors, therefore these behaviors and physical activity were combined into one category. Similarly, interventions and policies targeting narrowly defined dietary behaviors (e.g., a reduction of snacking) as well as addressing more complex dietary changes (e.g., a meal composition) were coded as referring to dietary behavior.

The *characteristics were allocated into 3 domains* proposed by the WHO [14]. They were considered as representing (1) main intervention/policy characteristics, (2) monitoring and evaluation processes, or (3) implementation issues. The allocation was conducted by 2 researchers (KH, MH) and verified by a third researcher (AL).

Next, characteristics within each domain were combined into *broader categories*. Two researchers (KH, AL) independently clustered all identified characteristics into categories. The names of categories and characteristics were then independently evaluated by the 3 researchers (MH, MvdB, GR) who searched for flaws in categorization and evaluated the meaningfulness of categories and characteristics.

Finally, the characteristic was *categorized as a good practice characteristic* if the respective characteristic was indicated in *either at least 4 systematic reviews or in at least 3 stakeholders’ documents*. This threshold is based on the number of documents supporting each characteristic and it represents the top quartile in the number of the supporting systematic reviews and stakeholders’ documents, respectively. To reach this threshold, the number of documents supporting a characteristic had to fall into the respective upper quartile: across characteristics, 75% were supported by 0–3 systematic reviews, whereas 25% were supported by at least 4 systematic reviews. Further, 75% characteristics were indicated in 0–2 stakeholder documents, whereas only 25% were indicated in at least 3 stakeholders’ documents (see Additional file 1). This arbitrary inclusion threshold was obtained in a consensus meeting by 3 research groups, represented by the researchers from 3 countries, involved in DEDIPAC project. The upper quartile-based thresholds are used in health promotion research eliciting good practice examples [22]. The threshold applied in the present study was considered as indicating *strong support* in analyzed data.

All good practice characteristic listed in Tables 1, 2, 3, 4 met this threshold (was supported by either at least 4 systematic reviews or by at least 3 stakeholders’ documents); 96 remaining characteristics (see Additional file 1) fell below the threshold and therefore were not included into the final list of best practice characteristics. For example, a good practice characteristic referring to the identification of environmental barriers and resources for participation was supported by 2 documents and therefore it was excluded from the final list.

**Table 1:** **The domain of main characteristics of good practice for interventions and policies aiming at dietary behavior and physical activity change**

**Good practice category**	**Systematic reviews, stakeholders’ documents, and position review papers endorsing respective characteristics**
Good practice characteristics	
**The use of theory**	
Theory applied in the development of intervention/policy	*Systematic reviews* [6,23-42]; *Stakeholders’ documents* [14,43-46]; *Position reviews* [47-49].
**Participants**	
Target audience well defined (including socio-demographic characteristics, risk factors, and susceptibility factors)	*Systematic reviews* [21,24-26,31,39,50-59]; *Position reviews* [60,61].
Needs of target group are identified (needs are assessed; they inform the content of intervention/policy; target group involved in policy/intervention development)	*Systematic reviews* [21]; *Stakeholders’ documents* [14,46,62,63].
Family involvement (parents participating in programs for children/adolescents)	*Systematic reviews* [24,30,37,50,55,64-69]; *Stakeholders’ documents* [43,70,71]; *Position reviews* [47,49,72].
**Target behavior**	
Target behavior well defined, specified, and adjusted to target population (e.g., walking, not physical activity)	*Systematic reviews* [36,37,39,41,50,51,57,66,68,73-75]; *Stakeholders’ documents* [14,63,76]; *Position reviews* [49,77-79].
**Multidimensional approach**	
Multidimensionality of the approach (e.g., addressing individual/personal factors, social, and physical environment)	*Systematic reviews* [3,29,56,68,80-82]; *Stakeholders’ documents* [14,62,70,71,76,83-85]; *Position reviews* [72,86-88].
Physical environment accounted for (environmental structures, transportation, land use, etc.)	*Systematic reviews* [3,34,50,64]; *Stakeholders’ documents* [71]; *Position reviews* [86,89].
**Content development and content management**	
Individual contacts and its intensity specified (including intensity of individual contacts with practitioners delivering interventions)	*Systematic reviews* [23,27,28,36,50,52,80,90-93]; *Stakeholders’ documents* [43,84]; *Position reviews* [8].
Duration (number of sessions, their length, frequency)	*Systematic reviews* [24,27,28,31,36,37,51-53,55,69,73,74,92,94-98].
Form of delivery (short messages, web based, self-guided with or without human support)	*Systematic reviews* [25,27,28,31-33,39,42,50,52,56,73,74,96,99-103]; *Stakeholders’ documents* [46,72].
Number of components (distinguishable elements/strategies used to prompt healthy diet/physical activity)	*Systematic reviews* [34,42,58,68,74,81,82,90,98]; *Stakeholders’ documents* [76,84].
General use of behavior change techniques: The use of any theory-based behavior change techniques	*Systematic reviews*: [6,23-26,30,31,33-35,37,38,40-42,50,73,97,98,104,105]; *Stakeholders’ documents* [43,71,76,106]; *Position reviews* [47,61,77].
Clarity achieved (clear presentation of the content, aims, processes, relations between elements, objectives)	*Systematic reviews* [21]; *Stakeholders’ documents* [43,45,62].
Tailoring (the content or materials adjusted to key characteristics of a target group)	*Systematic reviews* [24,27,32,51,54,56,80,90,97,99,101,107]; *Stakeholders’ documents* [62,106]; *Position reviews* [77].
Manuals/exact protocols exist (exact descriptions of content, components, and schedule of intervention/policy)	*Systematic reviews* [52]; *Stakeholders’ documents* [44,45,62,63].
The use of specific behavior change techniques: Self-monitoring and self-management strategies	*Systematic reviews* [6,26,41,104,108,109].
**Practitioner and setting contexts**	
Practitioners well defined (skills, training, and required characteristics specified)	*Systematic reviews* [25,26,29,31,52,59,73,98,102]; *Stakeholders’ documents* [83]; *Position reviews* [47,79].
Setting characteristics well defined	*Systematic reviews*: [23-25,31,32,34,39,50,52,55,56,58,59,64,65,68,98,103,110]; *Stakeholders’ documents: *[44,46,70]; *Position reviews*: [8,79,87].

**Table 2:** **The monitoring and evaluation domain of good practice characteristics for interventions and policies aiming at dietary behavior and physical activity change**

**Good practice category**	**Systematic reviews, stakeholders’ documents, and position reviews endorsing respective characteristics**
Good practice characteristics	
**Costs and funding**	
Costs in relation to obtained general health benefits (including population health changes, morbidity, quality of life, etc.)	*Systematic reviews* [111-113]; *Stakeholders’ documents* [46,76,114].
Costs related to behavior change (e.g., costs of an hour of PA gained per person)	*Systematic reviews* [39,51,95,107,111,112,115]; *Stakeholders’ documents* [46,76,114]; *Position reviews* [116].
Total financial costs of interventions/policies (total budget per participant)	*Systematic reviews* [53,95,101,111,112,115,117]; *Stakeholders’ documents* [45]; *Position reviews* [8,48,60,78];
**Outcomes**	
Outcomes measured with valid, reliable, and sensitive tools	*Systematic reviews*: Indicated in all included systematic reviews; *Stakeholders’ documents* [14,44,62].
Effects specified as clinically significant (e.g., moving from sedentary to physically active)	*Systematic reviews* [53,57,67,96,113,117]; *Stakeholders’ documents* [10,14,45]; *Position reviews* [47,79,116].
Effects on public health-relevant secondary outcomes (proximal, e.g., weight loss, and distal, e.g., heart disease morbidity)	*Systematic reviews* [55,59,74,81,111]; *Stakeholders’ documents* [14,45,46,118,119];
Negative consequences (or risks) evaluated	*Systematic reviews* [59,94,101,111,117,120]; *Stakeholders’ documents* [14,45,106,119]; *Position reviews*: [8,77,78].
Measured outcomes include physiological risk factor indices (e.g., BMI, cholesterol)	*Systematic reviews* [32,68,99,104,121].
**Effects’ evaluation: time and effect size**	
Efficiency established and reported (significant effects established in prior trials)	*Systematic reviews* Indicated in all included systematic reviews; *Stakeholders’ documents* [44,114,119]; *Position reviews* [107].
Sustainable effects (mid-term effects [>6 months] and long term effects [>12 months])	*Systematic reviews* [53,55,59,93-95,99,100,102,104,120,122]; *Stakeholders’ documents* [10,14,106,118,119]; *Position reviews* [8,47,86].
Effect sizes (besides significant effects)	*Systematic reviews* [23,28,31,36,75,92,99,122]; *Stakeholders’ documents* [44,45,63,63 separate for intervention and policies]; *Position reviews* [60,88].
**Reach**	
Reach (the strategy is likely to involve a large percentage of the target population; reaching entire target population)	*Systematic reviews* [53,59,94,100,107,112,117,123]; *Stakeholders’ documents* [10,44,63,83,118,119]; *Position reviews* [8,26,48,86].
Inclusiveness: health, age, and gender contexts (individuals with low mobility or comorbidities participate; including people of different age within target group)	*Systematic reviews* [53,59,94,100,107,112,117,123]; *Stakeholders’ documents* [14,44,45,46,62,63,63 (separate entries for intervention and policies), 106]; *Position reviews* [47,87].
Cultural competence and social inclusion of interventions/policies (accounts for cultural/minority issues in: recruitment processes, content, setting; familiarity with health practices in respective social/cultural groups)	*Systematic reviews* [27,29,40,53,59,94,100,101,109,112,117,123]; *Stakeholders’ documents* [43,44,76,83,119,124]; *Position reviews* [49,61].
**Participation and generalizability of evaluation**	
Generalizability of effects evaluated (effects observed among participants with different characteristics; effects at population level)	*Systematic reviews* [53,67,68,94]; *Stakeholders’ documents* [118,119].
Participation rates reported (across stages of evaluation)	*Systematic reviews* [39,53,96,117,120]; *Stakeholders’ documents* [14,44,118,119]; *Position reviews* [8].
**Underlying processes and active components**	
Active components identified	*Systematic reviews* [6,23,29,38,111]; *Position reviews* [88].
Ongoing monitoring and measurement of delivery and monitoring of materials	*Systematic reviews* [59,96,120]; *Stakeholders’ documents* [14,46,63,85].

**Table 3:** **The implementation domain of good practice characteristics for interventions and policies aiming at dietary behavior and physical activity change**

**Good practice category**	**Systematic reviews, stakeholders’ documents, and position reviews endorsing respective characteristics**
Good practice characteristics	
**Participation processes**	
Completion, attrition rates across stages (and their representativeness)	*Systematic reviews*: [53,59,75,90,100]; *Stakeholders’ documents:* [14,118]; *Position reviews*: [8].
Resources and strategies for practitioners helping them to invite and follow-up participants	*Systematic reviews*: [3,67,80,125]; *Stakeholders’ documents:*[63,106].
Strategies promoting long-term participation (maintenance) included	*Systematic reviews*: [26,47,64,80,93,97].
**Training for practitioners**	
Training for staff in aspects of implementation and facilitation of inter-sectorial collaboration	*Systematic reviews*: [3,53,59,95]; *Position reviews*: [8]
**Use/integration of existing resources**	
Resources for implementation specified	*Stakeholders’ documents:* [62,63,119].
Implementation integrated into existing programs (available for target population)	*Systematic reviews*: [112]; *Stakeholders’ documents:* [14,85,118,119].
Ongoing support from support from stakeholders secured	*Stakeholders’ documents:* [14,45,72,124].
**Feasibility**	
Adoption by target staff, settings, or institutions (representativeness of staff, settings, institutions; exclusion of settings, staff, institutions; characteristics of those who adopted vs those who did not)	*Systematic reviews*: [39,94,100,117]; *Stakeholders’ documents:* [44,118].
Feasible/acceptable for providers (fitting their skills; no external specialists needed for implementation), feasible and acceptable for stakeholders, and participants	*Systematic reviews*: [26,29,39,94,112]; *Position reviews*: [47,48,78,88].
**Maintenance-sustainability**	
Maintenance (effects maintained over time with institutional support; continuation within the realm of the institution)	*Systematic reviews*: [39,94,112,117]; *Stakeholders’ documents:* [62,119].
Mutability (intervention/policy is in the realm of community/target group control)	*Stakeholders’ documents:* [10,44,62,118,119]; *Position reviews*:[8,48,88].
**Partnership for implementation**	
Partnership between agencies/organizations to facilitate adoption and implementation (e.g., school, business, transport agencies; inter-sectorial collaboration between stakeholders)	*Systematic reviews*: [3]; *Stakeholders’ documents:* [14,43,72,76,124].
Identification of those who are responsible for implementation; training, monitoring and feedback for those responsible for implementation	*Stakeholders’ documents:* [43,46,62,63,119].
**Implementation consistency and adaptation processes**	
Implementation consistency and adaptations made during delivery assessed	*Systematic reviews*: [39,94]; *Stakeholders’ documents:* [45,118,124]; *Position reviews*: [8].
Adherence to protocol and protocol fidelity monitored	*Systematic reviews*: [52,59,95,100]; *Position reviews*: [8].
**Transferability**	
Transferability (interventions/policies can be transferred to other populations, communities, settings, and cultures)	*Systematic reviews*: [29,112]; *Stakeholders’ documents:* [10,45,62,119].
Context of transfer and transfer boundaries (including political, social, or economical conditions for transfer)	*Stakeholders’ documents:* [44,45,119].

**Table 4:** **The checklist of good practice characteristics for healthy diet and physical activity interventions and policies**

**No.**	**Best practice characteristic**
	**Main intervention/policy characteristics**
1a	Target audience well defined
2a	Target group needs identified
3a	Family involvement*
4b	Target behavior well defined and adjusted to target population
5c	Multidimensionality of the approach (individual, social, environmental)
6c	Physical environment accounted for
7d	Theory applied in the development of the intervention/policy
8e	Individual contacts and their intensity specified
9e	Duration (number of sessions, their length, and frequency)
10e	Forms of delivery
11e	Number of components (distinguishable elements/strategies used to prompt healthy diet/physical activity)
12e	The use of any theory-based behavior change techniques
13e	Clarity achieved
14e	Tailoring content and materials
15e	Manuals/exact protocols exist
16e	The use of specific behavior change techniques: self-monitoring and self-management
17f	Practitioners well defined
18f	Setting characteristics well defined
	**Monitoring and evaluation**
19 g	Costs in relation to obtained general health benefits
20 g	Costs related to behavior change
21 g	Total financial costs of the interventions/policy
22 h	Outcomes measured with valid, reliable, and sensitive tools
23 h	Effects specified as clinically significant
24 h	Effects on public health-relevant secondary outcomes
25 h	Negative consequences (or risks) evaluated
26 h	Measured outcomes include physiological risk factor indices
27i	Efficiency established and reported
28i	Sustainable effects
29i	Effect sizes
30j	Reach
31j	Inclusiveness: health, age, and gender context
32j	Cultural competence and social inclusion of the intervention/policy
33 k	Generalizability of effects evaluated
34 k	Participation rates reported
35 l	Active components identified
36 l	Ongoing monitoring and measurement of delivery; monitoring of materials
	**Implementation**
37 m	Completion and attrition rates across stages
38 m	Resources/strategies for staff helping them to invite and follow participants up
39 m	Strategies promoting long-term participation (maintenance) included
40n	Staff training in implementation and facilitation of inter-sectorial collaboration
41o	Resources for implementation specified
42o	Implementation integrated into existing programs
43o	Ongoing support from stakeholders secured
44p	Adoption by target staff, settings, or institutions
45p	Feasible/acceptable for providers, stakeholders, and participants
46q	Maintenance (the policy/intervention is maintained over time with institutional support)
47q	Mutability (the intervention/policy is in the realm of community/target group)
48r	Partnership between agencies/organizations to facilitate adoption/implementation
49r	Identification of those responsible for implementation; training and feedback for implementers
50s	Implementation consistency and adaptations made during delivery assessed
51 t	Adherence to protocol/protocol fidelity monitored**
32u	Transferability
53u	Contexts of transfer and transfer boundaries

Note: ‘a’ to ‘u’ represent 20 categories of best practice characteristics; * - characteristics identified mainly in documents referring to interventions/policies for children and adolescents; ** - characteristics identified mainly in documents referring to interventions.

## Results

### Description of analyzed material

The final selection included 74 systematic reviews (67.9%), 19 stakeholders’ documents (17.4%) and 16 position review papers (14.7%). Systematic reviews investigated a total of 2989 original studies. Populations analyzed in original papers included: general population samples (*k* = 31, 28.4%), children (*k* = 21, 19.4%), adolescents (*k* = 2, 1.8%), children and adolescents (*k* = 13, 11.9%), adults (*k* = 14, 12.8%), adults with a chronic disease, including cardiovascular or neurological diseases, diabetes, depression, obesity (*k* = 15, 13.8%), pregnant women (*k* = 1, 0.9%), adults at workplace (*k* = 6, 5.5%), older adults (*k* = 5, 4.7%), and vulnerable populations, such as ethnic minorities (*k* = 1, 0.8%). Three documents focused on women only. The majority of documents (*k* = 64, 58.7%) provided recommendations which could be applied both in policies and interventions, 39 (35.8%) formulated recommendations for interventions only; 6 (5.5%) focused on policy only. The majority referred to both physical activity and dietary behaviors (*k* = 62, 56.9%), whereas 36 (33%) analyzed dietary behaviors only and 11 (10.1%) addressed physical activity/sedentary behaviors only. The majority of documents (*k* = 97; 89%) referred to multi-level interventions and policies (i.e., using techniques aiming at a change at individual and social/environmental levels). Additional file 1 yields a description of original documents.

Quality of papers included into analysis ranged from moderate to minimal flaws (see Additional file 1). For systematic reviews, MQC scores ranged from 4 to 7, *M* = 5.50, *SD* = 0.93. In case of stakeholders’ documents and position review papers the scores for MQC-based measure ranged from 4 to 6, *M* = 4.68, *SD* = 0.60.

### Good practice attributes

Regarding *main intervention/policy characteristics*, we identified 40 good practice characteristics that were reported in at least one document (see Additional file 1). The characteristics were grouped into 6 distinct categories: the use of theory (*n* = 1), participants, (*n* = 6), target behavior (*n* = 6), content development and content management (*n* = 16), multidimensionality of interventions/ policies (*n* = 4), practitioner and setting contexts (*n* = 7).

Strong support was found for 18 good practice characteristics. They were reported in at least 4 systematic reviews or at least 3 stakeholders’ documents and thus coded as good practice characteristics (Table 1). The list of main intervention/policy characteristics includes: 1 attribute referring to the use of theory, 3 for participants, 1 for target behavior, 9 for content development and content management, 2 for multidimensionality of intervention/policy, and 2 for practitioner and setting contexts (Table 1). The majority of good practice characteristics (17 out of 18) were generic, that is they were indicated in documents referring to both diet and physical activity/sedentary behavior, referred to different age groups, interventions, and policies (see Additional file 1). The exception is ‘family involvement’, a characteristic referring to interventions and policies targeting children and adolescents only.

The analysis of original documents yielded 37 *monitoring and evaluation* characteristics of good practice (see Additional file 1). The characteristics were grouped into 6 categories: costs and funding (*n* = 5), outcomes (*n* = 11), the evaluation of effects: time and effect size (*n* = 6), reach (*n* = 5), the evaluation of participation and generalizability (*n* = 6), underlying processes and active components (*n* = 4).

Strong support was found for 18 good practice characteristics (Table 2), which represent the attributes of good practice referring to processes of monitoring and evaluation. They were indicated in at least 3 stakeholders’ documents or 4 systematic reviews. The list of good practice characteristics referring to monitoring and evaluation includes: 3 attributes referring to costs and funding, 5 for outcomes, 3 for the evaluation of effects: time and effect size, 3 for reach, 2 for the evaluation of participation and generalizability, and 2 for processes and active components. All 18 characteristics in the monitoring/evaluation domain were generic: they were found in documents referring to diet and physical activity/sedentary behavior, different age groups, interventions, and policies.

Finally, we identified 72 *implementation* good practice characteristics (see Additional file 1). They included: participation processes (*n* = 13), training for practitioners (*n* = 4), the use/integration of existing resources (*n* = 18), feasibility (*n* = 4), maintenance and sustainability (*n* = 8), partnership for implementation (*n* = 7), implementation consistency and adaptation processes (*n* = 13), and transferability (*n* = 5).

In contrast to findings for other domains (main characteristics and evaluations/processes), the majority of implementation characteristics (72%) was endorsed by less than 3 documents (Additional file 1). Only 17 met the threshold of strong support; only these characteristics were included into the final list of good practice characteristics. The final list includes: 3 attributes for participation processes, 1 for training for practitioners, 3 for use/integration of existing resources, 2 for feasibility, 2 for maintenance and sustainability, 2 for partnership for implementation, 2 for implementation consistency and adaptation processes, and 2 for transferability (see Table 3). The vast majority of good practice characteristics referring to implementation (16 out of 17) were generic. The exception was ‘adherence to protocol/protocol fidelity monitoring’ characteristic which was indicated in documents analyzing interventions, but not policies.

In sum, data synthesis yielded 149 good practice characteristics, referring to policies and interventions aiming at healthy diet and physical activity/sedentary behaviors. We found stronger support for 53 good practice characteristics, of which 51 are generic. The list of good practice characteristics was combined into a checklist (Table 4), which may be used for developing practice and reporting research on interventions and policies.

## Discussion and conclusions

This study provides an insight into good practice characteristic in interventions and policies targeting healthy diet, physical activity, and sedentary behavior in various populations. We identified 53 good practice attributes (51 generic), falling into 3 broad domains proposed by WHO [14]: main characteristics, monitoring/evaluations, and implementation. Across these domains, a similar number of characteristics of good practice was identified (18, 18, and 17, respectively), which may be an indicator of equivalent relevance of 3 domains.

Our efforts to identify characteristics which are evidenced and practice-based characteristics were undertaken in a response to concerns and appeals of practitioners, researchers, and editors which indicate difficulties in replicating and applying interventions/policies in various populations, because research reports present limited detail [4,7]. Compared to other lists [4,12] which serve similar purposes, the list of good practice characteristics developed in the present umbrella review was not restricted to the one domain of main descriptive intervention or policy characteristics, but also emphasizes the important domains of implementation and evaluation.

The list of 53 potentially crucial practice characteristics may be seen as a point of departure for further syntheses. The list might be shortened if future research would provide evidence for a lack of relevance of some characteristics for the success of interventions/policies. Until then, this broad list has a potential to inspire accumulating more detailed data and, in consequence, it would allow for identifying characteristics responsible for a success of interventions/policies.

The findings indicated that the majority of the 53 characteristics were endorsed by researchers as well as stakeholders, responsible for issuing practice recommendations. So far research on practice attributes reviewed either peer-reviewed studies [20,23] or stakeholders’ guidelines [21]. Combining 2 types of sources reinforces the conclusions. The list proposed in the present study integrates findings and concerns of researchers, practitioners, and those responsible for developing practice guidelines.

In sum, the list of good practice characteristics provides a comprehensive overview of specific aspects of potentially successful interventions and policies. Researchers, practitioners and policy makers may account for those characteristics when planning, developing, and reporting interventions and policies promoting healthy diet and physical activity. Compared to other lists of guidelines for reporting interventions/policies, the present list is based on a systematic review of empirical evidence and stakeholders’ proposals, therefore it may be feasible not only for researchers, but also for practitioners who need to apply the guidelines formed by major national stakeholders. Further, compared to other proposals, our list does not focus on broad categories e.g. [8], which may be difficult to translate into practice, but on specific, narrowly defined characteristic. Based on existing evidence it may be assumed that accounting for these characteristics increases the likelihood of developing a successful policy or intervention.

We analyzed characteristics indicated in position papers, which presented attributes of practice based on non-systematic reviews of literature. Although those papers were of relatively high quality, the support for a practice characteristic found in this type of documents was not used as a criterion for including the characteristic into the final list of attributes of good practice. A relatively small number of characteristics from the preliminary list (18 out of 149) was supported solely by position papers. It has to be noted, that in the domain of implementation the number of characteristics indicated in position papers only was twice as high as in 2 remaining domains. Therefore, future research investigating implementation practices should explore evidence accumulated in systematic and non-systematic reviews.

Our study has several limitations. The proposed list of good practice characteristics is based on an umbrella review of reviews and stakeholders’ documents. Thus, some recent studies on good practice characteristics were not included. Furthermore, the present study did not differentiate between target groups (e.g., adults versus children) and it is possible that some good practice characteristics are more relevant for some target groups than for others. Additionally, we combined evidence for good practice characteristics for policies and interventions. Although 52 out of 53 characteristics were supported in documents referring to both interventions and policies, future research should investigate if attributes of good practices are different for policies and for interventions. The decision to define characteristics as the attributes of good practice was based on an arbitrary criterion (i.e. the number of documents supporting the characteristic had to fall into the upper quartile for the number of either systematic reviews or stakeholders’ documents), which was chosen by a consensus method [17]. As indicated, the quartile-based thresholds are used in health promotion research eliciting good practice examples [22]. The main limitation of this approach refers to the fast progress in accumulation of the evidence: as new systematic reviews and stakeholders’ documents are published every year, the characteristic that just missed the threshold may fit the criteria of good practice characteristics very soon. Further, with growing evidence the threshold may need to be changed as a different number of documents would represent the upper quartile. Therefore, as new evidence is accumulating, this list should be updated regularly. Finally, the evaluation of the quality of the material included in the present study was based on criteria which were relatively lenient. In sum, all conclusions should be treated with caution and the proposed list of characteristics is preliminary.

In conclusion, our study provides a broad list of good practice characteristics in interventions and policies targeting healthy diet and physical activity. Research aiming at defining successful interventions and policies may need to report the presence (and, where feasible, the content) of those characteristics. The use of the proposed list of good practice characteristics may foster further development of health promotion sciences, as it would allow for identification of success vectors in the domains of main characteristics of interventions/policies, their implementation, evaluation and monitoring processes.

## Additional file

Additional file 1:
**Quality evaluation criteria for stakeholders’ documents, descriptive data for all reviewed documents and the list of 149 elicited characteristics (with supporting documents).** Additional file includes: (a) the quality evaluation criteria for stakeholders’ documents Methodological Quality Checklist for Stakeholders’ Documents and Position Papers; (MQC-SP); (b) descriptive data retrieved from systematic reviews, stakeholder documents and position review papers included into the umbrella review, (c) the list of 149 best practice characteristics and references to the documents supporting the characteristics.

## Abbreviations

DEDIPAC: The Knowledge Hub on the DEterminants of DIet and Physical ACtivity
MQC: Methodological Quality Checklist
