# Supplementary material for: Good practice characteristics of diet and physical activity interventions and policies: an umbrella review
Source: BMC Public Health. 2015 Jan 21;15:19. doi: 10.1186/s12889-015-1354-9 (PMC4306239; doi:10.1186/s12889-015-1354-9)
Supplement: Additional file 1: — Quality evaluation criteria for stakeholders’ documents, descriptive data for all reviewed documents and the list of 149 elicited characteristics (with supporting documents). Additional file includes: (a) the quality evaluation criteria for stakeholders’ documents Methodological Quality Checklist for Stakeholders’ Documents and Position Papers; (MQC-SP); (b) descriptive data retrieved from systematic reviews, stakeholder documents and position review papers included into the umbrella review, (c) the list of 149 best practice characteristics and references to the documents supporting the characteristics. [file 12889_2015_1354_MOESM1_ESM.docx]

***Additional file 1***

*Table S1: Methodological Quality Checklist for Stakeholders’ Documents and Position Papers (MQC-SP)*

| Criteria and their description | Scoring |
| --- | --- |
| **1. Is there a major stakeholder involved?**  - The document is developed/endorsed by (1) a nation-wide or international organization which is issuing recommendations and guidelines which are used in clinical practice; or (2) an interdisciplinary or cross-country consortium aiming at providing progress in the discipline/practice for respective behavior | 0 (no) or 1 (yes) |
| **2. Is there a well-defined aim?**  – The document specifies the aim of the paper, target population, the type of actions and their breadth (e.g., changes in physical environment, any school-based interventions and policies) and the type of relevant behavior | 0 (no) or 1 (yes) |
| **3. Is there a robust methodology?**  – The method should list the sources used to obtain *comprehensive and heterogeneous* data, such as literature review and analysis of several examples of interventions/policies, and “grey literature” or unpublished documents | 0 (no) or 1 (yes) |
| **4. Quality evaluation of analyzed material applied?**  – The document refers to the quality evaluation of the included material and/or refers to quality evaluation methods or measures | 0 (no) or 1 (yes) |
| **5. Have the included material been appropriately synthesized?**  – The synthesis of analyzed material addresses the heterogeneity of analyzed data; provides specific conclusions; conclusions are supported by analyzed material; the key constructs are clearly operationalized | 0 (no) or 1 (yes) |
| **6. Has more than one stakeholder/author been involved at the process?**  – To minimize bias, conclusions were based on involvement and consensus achieved by at least two stakeholders/ multiple researchers from different organizations | 0 (no) or 1 (yes) |
| **Total score: low = 0-3, moderate: 4-5; high 6** | 0-6 |

*Table 2. Description of systematic reviews, stakeholder documents and position review papers included into the umbrella review.*

| First author, year of publication | Type of the paper | Number of original studies | Design of original studies | Population | Behavior | Policy or intervention implications? | Multilevel or one level | Quality |
| --- | --- | --- | --- | --- | --- | --- | --- | --- |
| Aalbers 2011 | 1 | 12 | RCT, CT | Adults (older) | Diet and PA | Intervention | Multilevel | 5 |
| Ajie 2014 | 1 | 15 | RCT, CT | Adolescents | Diet | Intervention | Single level | 6 |
| Aldcroft 2011 | 1 | 7 | RCT, quasi-experimental | Adults (chronic disease) | Diet and PA | Interventions | Single level | 7 |
| America Dietetic Association 2006 | 2 | - | - | Children | Diet and PA | Both | Multilevel | 5 |
| American Dietetic Association 2003 | 2 | - | - | Children | Diet and PA | Both | Multilevel | 4 |
| American Dietetic Association 1999 | 2 | - | - | Children | Diet | Both | Multilevel | 4 |
| Anderson 2009 | 1 | 40 | RCT, Cluster RT, CT, Prospective cohort studies | Adults (at workplace) | Diet and PA | Both | Multilevel | 6 |
| Avery 2013 | 1 | 40 | RCT, prospective cohort studies | Adults | Diet | Interventions | Multilevel | 5 |
| Ayliffe 2010 | 1 | 63 | RCT, Cluster RT, CT, Prospective cohort studies | Children | Diet and PA | Both | Multilevel | 5 |
| Bellew 2008 | 3 | - | - | General population | PA | Policy | Multilevel | 5 |
|  |  |  |  |  |  |  |  |  |
| Besculides 2008 | 3 | - | - | Adults (women) | Diet and PA | Both | Multilevel | 5 |
| Biddle 2014 | 1 | 10 | Systematic review | Children and Adolescents | PA | Interventions | Multilevel | 5 |
| Bock 2013 | 1 | 55 | RCT; quasi experimental | General population | PA | Both | Multilevel | 5 |
| Bossen 2014 | 1 | 7 | RCT, Cluster RT, CT, Prospective cohort studies | Adults (chronic disease) | PA | Intervention | Single level | 6 |
|  |  |  |  |  |  |  |  |  |
| Brennan Ramirez 2006 | 3 | - | - | General population | PA | Policy | Multilevel | 5 |
| Brennan 2014 | 1 | 396 | Any quantitative study; any review | Children | Diet and PA | Both | Multilevel | 4 |
| Broekhuizen 2012 | 1 | 50 | RCT | Adults | Diet and PA | Intervention | Multilevel | 5 |
| Brown 2012 | 1 | 16 | RCT, quasi-experimental, cross-sectional, prospective cohort | General population | PA | Both | Single level | 5 |
| Burrows 2012 | 1 | 31 | RCT, CT | Children | Diet | Both | Multilevel | 6 |
| Campbell 2007 | 1 | 9 | RCT, CT, quasi-experimental, cluster CT | Children | Diet and PA | Both | Multilevel | 5 |
| Carroll 2011 | 1 | 38 | RCT, CT, quasi-experimental, cluster CT | Adults (primary care) | PA | Both | Multilevel | 5 |
| Choi 2013 | 1 | 11 | RCT | Adults (pregnant women) | Diet and PA | Intervention | Multilevel | 6 |
| Christiansen 2014 | 3 | - | - | General population | PA | Policy | Multilevel | 5 |
| Cress 2006 | 3 | - | - | Adults (aged 50+) | PA | Both | Multilevel | 4 |
| De Bourdeaudhuij 2011 | 1 | 11 | RCT, CT, cluster CT | Children and adolescents | Diet and PA | Intervention | Multilevel | 7 |
| De Meester 2009 | 1 | 20 | RCT, CT, cluster CT, quasi-experimental | Adolescents | PA | Both | Multilevel | 6 |
| Dixey 1999 | 2 | - | - | General population | Diet | Both | Multilevel | 4 |
| Dombrowski 2012 | 1 | 44 | RCT | Adults | Diet and PA | Intervention | Single level | 7 |
| Dubois 2008 | 2 | - | - | General population | Diet and PA | Both | Multilevel | 4 |
| Edwardson 2010 | 1 | 96 | Cross-sectional, longitudinal (correlational) | Children and adolescents | PA | Both | Multilevel | 5 |
| Flynn 2006 | 1 | 147 | RCT, CT, quasi-experimental, cohort, observational | Children | Diet and PA | Both | Multilevel | 7 |
| Foltz 2012 | 3 | - | - | Children and adolescents | Diet and PA | Both | Multilevel | 4 |
| Geaney 2013 | 1 | 6 | RCT, CT | Adults (at workplace) | Diet | Both | Multilevel | 6 |
| Geraedts 2013 | 1 | 32 | RCT, CT, quasi-experimental observational, | Adults (older) | PA | Both | Multilevel | 7 |
| Ghisi, 2014 | 1 | 42 | RCT, CT, quasi-experimental, observational, cohort, cross-sectional, correlational-longitudinal | Adults (with chronic disease) | Diet and PA | Intervention | Multilevel | 6 |
| Gillison 2012 | 3 | - | - | General population | Diet and PA | Both | Multilevel | 5 |
| Glasgow 2007 | 3 | - | - | General population | Diet and PA | Both | Multilevel | 5 |
| Golley 2011 | 1 | 17 | RCT, CT, quasi-experimental observational, | Children and parents | Diet | Intervention | Multilevel | 5 |
| Goode 2012 | 1 | 25 | RCT, CT, observational | General population | Diet and PA | Intervention | Single level | 6 |
| Greaves 2011 | 1 | 30 | Systematic reviews | Adults | Diet and PA | Intervention | Single level | 7 |
| Gudzune 2013 | 1 | 5 | RCT, CT | Adults (college and worksite context) | Diet and PA | Intervention | Multilevel | 5 |
| Hamel 2013 | 1 | 15 | RCT, CT, | Children and adolescents | Diet and PA | Both | Multilevel | 6 |
| Hardeman 2000 | 1 | 9 | RCT, CT | General population | Diet and PA | Intervention | Multilevel | 5 |
| Haughton 2012 | 2 | - | - | Adults (older) | Diet | Both | Multilevel | 4 |
| Hearn 2008 | 1 | 45 | RCT, CT, quasi-experimental, observational | Children | Diet and PA | Both | Multilevel | 4 |
| Heath 2006 | 1 | 106 | Any qualitative | General population | PA | Both | Multilevel | 4 |
| Heath 2012 | 1 | 100 | Reviews (systematic and non-systematic) | General population | PA | Interventions | Multilevel | 5 |
| Hendrie 2012 | 1 | 15 | RCT, CT, quasi-experimental | Children | Diet and PA | Interventions | Multilevel | 5 |
| Hoehner 2013 | 1 | 19 | RCT, CT, quasi-experimental | General population | PA | Both | Multilevel | 7 |
| Hoelscher 2013 | 2 | - | - | Children | Diet and PA | Both | Multilevel | 6 |
| Husebo 2013 | 1 |  | RCT, CT, observational | Adults (chronic disease) | PA | Interventions | Multilevel | 6 |
| IMPALA 2011 | 2 | - | - | General population | PA | Policy | Multilevel | 4 |
| Kahn 2002 | 3 | - | - | General population | PA | Both | Multilevel | 6 |
| Katz 2005 | 3 | - | - | Children and adults (school and workplace context only) | Diet and PA | Both | Multilevel | 4 |
| Katz 2008 | 1 | 19 | RCT, CT, prospective cohort | Children and adolescents | Diet and PA | Both | Multilevel | 5 |
| Khan 2009 | 2 | - | - | Children/Adolescents | Diet and PA | Both | Multilevel | 6 |
| King 2011 | 3 | - | - | General population | Diet and PA | Both | Multilevel | 5 |
| Kirk 2012 | 1 | 19 | Systematic reviews, non-systematic reviews | Adult (chronic disease) | Diet and PA | Interventions | Multilevel | 4 |
| Klesges 2008 | 1 | 19 | RCT, CT | Children | Diet and PA | Both | Multilevel | 5 |
| Klesges 2012 | 1 | 77 | RCT CT | Children (with chronic disease) | PA and diet | Both | Multilevel | 5 |
| Kohl 2013 | 1 | 41 | Systematic reviews | Adults | Diet and PA | Intervention | Single level | 6 |
| Kriemler 2011 | 1 | 24 | Systematic reviews, RCT, CT | Children and adolescents | PA | Both | Multilevel | 5 |
| Latimer 2010 | 1 | 22 | RCT, CT | Adults | PA | Both | Multilevel | 6 |
| Lee 2010 | 1 | 27 | RCT | Adults (chronic disease) | PA | Both | Multilevel | 7 |
|  |  |  |  |  |  |  |  |  |
| Lombard 2009 | 1 | 9 | RCT, CT | Adults | Diet and PA | Both | Multilevel | 5 |
| Marsh 2014 | 1 | 17 | RCT, CT | Children and adolescents | PA | Both | Multilevel | 6 |
| Martin 2013 | 1 | 17 | RCT | Children | Diet and PA | Interventions | Single level | 5 |
| McNeil 2006 | 3 | - | - | General population | Diet and PA | Both | Multilevel | 4 |
| Michie 2009 | 1 | 122 | RCT, CT, cluster trials, other intervention studies with control group | Adults | Diet and PA | Intervention | Single level | 5 |
| Morris 2014 | 1 | 11 | RCT, CT | Adults (chronic disease) | PA | Intervention | Multilevel | 6 |
| Müller-Riemens chneider, 2008 | 1 | 25 | RCT | Adults | PA | Intervention | Multilevel | 5 |
| Nawaz 2001 | 2 | - | - | Adults (with chronic disease) | Diet and PA | Both | Multilevel | 4 |
| Netherlands Centre Youth Health 2013 | 2 | - | - | General population | Diet and PA | Both | Multilevel | 4 |
| Niemeier 2012 | 1 | 42 | RCT, CT | Children and adolescents | PA | Both | Multilevel | 5 |
| Nixon 2012 | 1 | 12 | RCT, CT, cluster CT | Children | Diet and PA | Interventions | Multilevel | 6 |
| Ogilvie 2007 | 1 | 48 | RCT, CT | General population | PA | Interventions | Multilevel | 4 |
| Paul-Ebhohimhen 2009 | 1 | 5 | RCT | Adults(chronic disease) | Diet and PA | Both | Multilevel | 5 |
| Pearson 2012 | 1 | 18 | RCT | Adults (with chronic disease) | Diet and PA | Intervention | Multilevel | 5 |
| Pratt 2008 | 2 | - | - | Children | Diet and PA | Both | Multilevel | 4 |
| Prestwich 2014 | 1 | 190 | RCT, CT | Adults | Diet and PA | Intervention | Single level | 6 |
| Rabin 2010 | 1 | 7 | Cluster RT, cross-sectional, observational | General population | PA an Diet | Interventions | Multilevel | 6 |
| NCI 2012 | 2 | - | - | General population | Diet and PA | Both | Multilevel | 4 |
| Richardson 2013 | 1 | 19 | Published guidelines | Children | Diet and PA | Both | Multilevel | 6 |
| Robertson 2012 | 1 | 8 | RCT | Adults (chronic diseases | PA | Interventions | Multilevel | 6 |
| Roux 2008 | 1 | 7 | RCT, CT | Adults | PA | Both | Multilevel | 4 |
| Salmon 2007 | 1 | 57 | RCT, CT, cluster CT, quasi-experimental, observational | Children and adolescents | PA | Both | Multilevel | 4 |
| Schröer 2014 | 1 | 15 | Systematic reviews | Adults (at workplace) | Diet and PA | Both | Multilevel | 5 |
| Simovska, 2010 | 2 | - | - | Children and Adolescents | Diet and PA | Policy | Multilevel | 4 |
| Spencer 2013 | 2 | - | - | General population | Diet and PA | Both | Multilevel | 4 |
| Stephens 2014 | 1 | 83 | Systematic Reviews | General population | Diet and PA | Both | Multilevel | 6 |
| Stockley 2001 | 2 | - | - | General population | Diet | Both | Multilevel | 4 |
| Sumlin 2012 | 1 | 15 | RCT, quasi-experimental | Adults (women with chronic disease) | Diet | Both | Multilevel | 4 |
| Summerbell 2012 | 3 | - | - | Children | Diet and PA | Both | Multilevel | 5 |
| Swinburn 2005 | 3 | - | - | General population | Diet and PA | Policy | Multilevel | 5 |
| Taylor 2007 | 1 | 38 | Systematic reviews | Adults (chronic disease) | PA | Both | Multilevel | 7 |
| Thomson 2011 | 1 | 34 | RCT | General population | Diet | Intervention | Multilevel | 5 |
| Tierney 2012 | 1 | 9 | RCT | Adults (with chronic disease) | PA | Interventions | Multilevel | 6 |
| Trudnak 2012 | 3 | - | - | Children | Diet and Pa | Both | Multilevel | 4 |
| van Achterberg 2011 | 1 | 9 | Systematic reviews | Adults (chronic disease) | Diet and PA | Interventions | Multilevel | 7 |
| van Sluijs 2008 | 1 | 57 | RCT, CT | Children and adolescents | PA | Interventions | Multilevel | 7 |
| Vuillemin 2011 | 1 | 33 | RCT, CT, cluster CT, observational, quasi-experimental, | Adults (at workplace) | PA | Intervention | Multilevel | 7 |
| Walton-Moss 2013 | 1 | 32 | RCT, quasi-experimental, observational | Vulnerable populations (e.g., minority, low SES) | Diet and PA | Interventions | Multilevel | 4 |
| Weaver 2012 | 3 | - | - | Children and adolescents | Diet and PA | Both | Multilevel | 4 |
| Webb 2010 | 1 | 85 | RCT, CT, other with control group | Adults | Diet and PA | Intervention | Single level | 5 |
| Weightman 2005 | 2 | - | - | General population | Diet and PA | Interventions | Multilevel | 5 |
| Wilding 2013 | 1 | 42 | RCT, CT, systematic reviews | Adults (older) | PA | Both | Multilevel | 4 |
| Wu 2011 | 1 | 91 | RCT, CT, quasi-experimental, observational, cohort | General population | PA | Both | Multilevel | 6 |
| WHO 2011 | 2 | - | - | General population | Diet and PA | Both | Multilevel | 4 |
| WHO 2013 | 2 | - | - | General population | PA | Separate recommendations for policies/interventions | Multilevel | 4 |

*Note*: Types of documents: 1 - systematic reviews, 2 - stakeholder documents, 3 - position review papers; PA – physical activity; RCT – randomized controlled trail; CT – controlled trial; Observational: studies with pre- and post-test for intervention group (no control group); Quasi experimental – studies without full evaluation of pre- and post-tests in both control and intervention group(s); Chronic diseases: obesity, cardiovascular diseases, neurological diseases, depression, diabetes, cancer; Quality – total scores for MQC (systematic reviews) or MQC-SP (for stakeholders’ documents and position review papers).

*Table 3: 149 Best practice characteristics and their documentation: main characteristics of intervention and polices*

| Best practice category  Best practice characteristics |  | | | Behavior | | | | Population | | | | | | | | | | Intervention/ policy | | | | | | | | | | | | | Number of levels | | | | | Systematic reviews, stakeholders’ documents, and position reviews endorsing respective characteristic | |
| --- | --- | --- | --- | --- | --- | --- | --- | --- | --- | --- | --- | --- | --- | --- | --- | --- | --- | --- | --- | --- | --- | --- | --- | --- | --- | --- | --- | --- | --- | --- | --- | --- | --- | --- | --- | --- | --- |
|  | Type of docu ment | | | | PA and diet | pa | diet | general population | children adolescents | | | | adults | | | | | intervention and policy | | | | | intervention | | | | policy | | | | Multi level | | | one level | |  |  |
| *The use of theory* | |  | | | | | | | | | | | | | | | | | | | | | | | | | | | | | | | | | | | |
| Theory applied for intervention/policy development | | 1 | | 14 | | 4 | 3 | 3 | | | | 6 | | | | | 12 | | | | 7 | | | | 14 | | | | |  | | 15 | | | 6 | | Bossen 2014; Lee 2010; Michie 2009; Rabin 2010; Prestwich 2014; Dombrowski 2012; Webb 2010; Greaves 2011; Tierney 2012; Avery 2013; Hendrie 2012;Nixon 2012; Husebo 2013; Ajie 2014; Bock 2013;Thomson 2011; Hardeman 2000; Golley 2011; Flynn 2006; Besculides 2008; Hamel 2013 |
|  | | 2 | | 4 | |  | 1 | 4 | | | | 1 | | | | |  | | | | 4 | | | | 1 | | | | |  | | 5 | | |  | | Stockley 2001; ADA ; 2003; WHO 2011; Dubois 2008; Netherlands Centre Youth Health 2013 |
|  | | 3 | | 3 | |  |  | 2 | | | | 1 | | | | |  | | | | 2 | | | | 1 | | | | |  | | 3 | | |  | | Summerbell 2012; Gillison 2012; McNeil 2006 |
| *Participation* | |  | | | | | | | | | | | | | | | | | | | | | | | | | | | | | | | | | | | |
| Protocol and recruitment tailored to a specific population if a target population is narrowed down (e.g. older adults) | | 1 | |  | | 2 |  | 1 | | |  | | | | | | 1 | | | | 2 | | | |  | | | | |  | | 1 | | | 1 | | Wilding 2013; Brown 2012 |
| Providing choice for participants | | 3 | | 1 | |  |  |  | | | 1 | | | | | |  | | | | 1 | | | |  | | | | |  | | 1 | | |  | | Weaver 2012 |
| Family involvement | | 1 | | 4 | | 6 | 1 |  | | | 11 | | | | | |  | | | | 8 | | | | 3 | | | | |  | | 10 | | | 1 | | Ayliffe 2010; Biddle 2014; Nixon 2012; Marsh 2014; de Meester 2009; Ajie 2014; Edwardson 2010; Golley 2011; Katz 2008; Kriemler 2011; Niemeier 2012; |
|  | | 2 | | 3 | | 1 |  |  | | | 4 | | | | | |  | | | | 4 | | | |  | | | | |  | | 4 | | |  | | Trudnak 2012; ADA 2006; ADA 1999; ADA 2003 |
|  | | 3 | | 2 | |  |  | 1 | | | 1 | | | | | |  | | | | 1 | | | | 1 | | | | |  | | 2 | | |  | | Summerbell 2012; Gillison 2012 |
| Environmental barriers and resources for participation identified | | 1 | | 1 | | 1 |  | 1 | | |  | | | | | | 1 | | | | 2 | | | |  | | | | |  | | 2 | | |  | | Besculides 2008; Heath 2012 |
| Target audience well defined (in terms of socio-demographics, risk factors, susceptibility factors) | | 1 | | 7 | | 7 | 2 | 1 | | | 7 | | | | | | 8 | | | | 10 | | | | 6 | | | | |  | | 13 | | | 3 | | Klesges 2012; Taylor 2007; Rabin 2010; Greaves 2011; Avery 2013; Biddle 2014; Marsh 2014; Salmon 2007; Latimer 2010; Ajie 2014; Richardson 2013; Brown 2012; Ghisi 2014; Besculides 2008; van Sluijs 2008; Vuillemin 2011 |
|  | | 3 | | 1 | | 1 |  | 2 | | |  | | | | | |  | | | | 1 | | | |  | | | | | 1 | | 2 | | |  | | Christiansen 2014; King 2011 |
| Target group needs identified (needs are assessed; they inform the content of intervention; target group involved in the policy/intervention development) | | 1 | |  | | 1 |  | 1 | | |  | | | | | |  | | | | 1 | | | |  | | | | |  | | 1 | | |  | | Richardson 2013 |
|  | | 2 | | 2 | | 1 | 1 | 2 | | | 2 | | | | | | - | | | | 3 | | | | - | | | | | - | | 4 | | | - | | WHO 2011; Dixey 1999; WHO 2013; Stockley, 2001 |
| *Target behavior* | |  | | | | | | | | | | | | | | | | | | | | | | | | | | | | | | | | | | | |
| Target behavior well defined and specified (e.g., walking, not PA), and adjusted to target population | | 1 | | 3 | | 8 | 1 | 1 | | | 5 | | | | | 6 | | | | 8 | | | | 4 | | | | |  | | | 12 | | | 1 | | Robertson 2012; Lee 2010; Taylor 2007; Rabin 2010; Burrows, 2012; Tierney 2012; Biddle 2014; Nixon 2012; Kirk 2012; Kriemler 2011; Brown 2012; Edwardson 2010; |
|  | | 2 | | 2 | | 1 |  | 2 | | | 1 | | | | |  | | | | 1 | | | | 2 | | | | |  | | | 3 | | |  | | Pratt 2008; WHO 2011; WHO 2013 |
|  | | 3 | | 2 | | 1 | 1 | 1 | | | 2 | | | | | 1 | | | | 3 | | | | 1 | | | | |  | | | 4 | | |  | | Summerbell 2012; Weaver 2012; Cress 2006; Kahn 2002 |
| Aims achievable and convincing | | 2 | |  | | 2 | 1 | 3 | | |  | | | | |  | | | |  | | | | 2 | | | | | 1 | | | 3 | | |  | | Dixey 1999 ; WHO 2013; WHO 2013 (Separate for intervention and policy) |
| Target relevant from public health perspective | | 2 | | 1 | |  |  | 1 | | |  | | | | |  | | | |  | | | | 1 | | | | |  | | | 1 | | |  | | WHO 2011 |
| Target behavior refers to the national guidelines | | 3 | | 1 | |  | 1 | 2 | | |  | | | | |  | | | | 1 | | | |  | | | | | 1 | | | 2 | | |  | | Bellew 2008; Glasgow 2007 |
| Target refers to common risk factors implicated in chronic diseases | | 1 | | 1 | |  |  |  | | | 1 | | | | |  | | | | 1 | | | |  | | | | |  | | | 1 | | |  | | Flynn 2006 |
| Individualized plans for behavior | | 1 | | 1 | |  |  |  | | | 1 | | | | |  | | | | 1 | | | |  | | | | |  | | | 1 | | |  | | Ayliffe 2010 |
| *Content development and content management* | |  | | | | | | | | | | | | | | | | | | | | | | | | | | | | | | | | | | | |
| Intensity of direct contact during intervention/policy | | 1 | | 4 | | 7 | 1 | 2 | | | 1 | | | | 9 | | | | 6 | | | | | 6 | | | | |  | | | 11 | | | 1 | | Bossen 2014; Lee 2010; Geraedts 2013; Choi 2013;Dombrowski 2012; Biddle 2014; Ghisi 2014; Müller-Riemenschneider 2008; Bock 2013; Carroll 2011; Aalbers 2011 |
|  | | 2 | | 2 | |  |  |  | | | 2 | | | |  | | | | 2 | | | | |  | | | | |  | | | 2 | | |  | | ADA 2003; Hoelscher 2013 |
|  | | 3 | | 1 | |  |  | 1 | | |  | | | |  | | | | 1 | | | | |  | | | | |  | | | 1 | | |  | | Glasgow 2007 |
| Duration of actions, number of sessions/actions, frequency of sessions/actions | | 1 | | 9 | | 9 | 2 | 4 | | | 7 | | | | 9 | | | | 13 | | | | | 7 | | | | |  | | | 16 | | | 4 | | Bossen 2014; Morris 2014; Lee 2010; Klesges 2012; Greaves 2011; Burrows, 2012; van Achterberg 2011; Nixon 2012; Lombard 2009; Kirk 2012; Ajie 2014; Brown 2012; Ghisi 2014; Goode 2012; Marsh 2014; Bock 2013; Brennan 2014; Niemeier 2012; Lee 2010; Geraedts 2013 |
| Forms of delivery (messages, web based, self-guided with or without human support) | | 1 | | 12 | | 6 | 1 | 5 | | | 4 | | | | 10 | | | | 8 | | | | | 11 | | | | |  | | | 16 | | | 3 | | Bossen 2014; Rabin 2010; Kohl 2013; Webb 2010; Greaves 2011; Broekhuizen 2012; Burrows, |
|  | |  | |  | |  |  |  | | |  | | | |  | | | |  | | | | |  | | | | |  | | |  | | |  | | 2012; Avery 2013; Biddle 2014; Lombard 2009; Paul-Ebhohimhen 2009; Kirk 2012; Ghisi 2014; Hardeman 2000; Hamel 2013; Salmon 2007; Ogilvie 2007; Bock 2003; Stephens 2014 |
|  | | 2 | | 1 | |  | 1 | 1 | | | 1 | | | |  | | | | 2 | | | | |  | | | | |  | | | 2 | | |  | | Trudnak 2012; Stockley 2001 |
| The number of components | | 1 | | 7 | | 2 |  |  | | | 3 | | | | 5 | | | | 4 | | | | | 5 | | | | |  | | | 8 | | | 1 | | Webb, 2010; Walton-Moss 2013; Hendrie 2012; van Achterberg 2011; Schröer 2014; Kirk 2012; Kriemler 2011; van Sluijs 2008; Aalbers 2011 |
|  | | 2 | | 2 | |  |  |  | | | 2 | | | |  | | | | 2 | | | | |  | | | | |  | | | 2 | | |  | | Pratt 2008; Hoelscher 2013 |
| Innovative (compared to other interventions/approaches available) | | 1 | |  | |  | 1 | 1 | | |  | | | |  | | | |  | | | | | 1 | | | | |  | | | 1 | | |  | | Heath 2012 |
|  | | 2 | | 2 | |  |  |  | | | 2 | | | |  | | | | 2 | | | | |  | | | | |  | | | 2 | | |  | | Hoelscher , 2013; Pratt 2008 |
|  | | 3 | | 1 | |  |  | 1 | | |  | | | |  | | | | 1 | | | | |  | | | | |  | | | 1 | | |  | | King 2011 |
| Multimodal techniques (interactivity) | | 1 | | 1 | | 1 |  | 1 | | | 1 | | | |  | | | | 1 | | | | | 1 | | | | |  | | | 2 | | |  | | Nixon 2012; Bock 2013 |
| The use of any theory-based behavior change techniques | | 1 | | 13 | | 4 | 5 | 2 | | | 7 | | | | 13 | | | | 3 | | | | | 19 | | | | |  | | | 13 | | | 9 | | Michie 2009; Aldcroft 2011; Prestwich 2014; Dombrowski 2012; Webb 2010; Greaves 2011; Burrows, 2012; Tierney 2012; Avery 2013; Biddle 2014; Hendrie 2012; van Achterberg 2011; Nixon 2012; Husebo 2013; Ajie 2014;Thomson 2011; Hardeman 2000; Golley 2011; Besculides 2008; Morris 2014; Martin 2013 |
|  | | 2 | | 4 | |  |  |  | | | 3 | | | | 1 | | | | 4 | | | | |  | | | | |  | | | 4 | | |  | | ADA 2006; Nawaz 2001; ADA 2003; Pratt 2008 |
|  | | 3 | | 2 | |  | 1 | 2 | | |  | | | | 1 | | | | 3 | | | | |  | | | | |  | | | 3 | | |  | | Cress 2006; Gillison 2012; King 2011 |
| Sequential programs (from preschool to high school) | | 2 | | 1 | |  |  |  | | | 1 | | | |  | | | | 1 | | | | |  | | | | |  | | | 1 | | |  | | ADA 2003 |
| Clarity of content, aims, and processes, relations between elements, objectives of intervention/policy | | 2 | | 2 | |  | 1 | 2 | | | 1 | | | |  | | | | 2 | | | | | 1 | | | | |  | | | 3 | | |  | | ADA 2003; Dixey 1999; Netherlands Centre Youth Health 2013 |
|  | | 1 | | 1 | |  |  |  | | | 1 | | | |  | | | | 1 | | | | |  | | | | |  | | | 1 | | |  | | Richardson 2013 |
| Simplicity of intervention/policy materials | | 3 | | 1 | |  |  |  | | | 1 | | | |  | | | |  | | | | | 1 | | | | |  | | | 1 | | |  | | Summerbell 2012 |
| Tailoring (the content and materials appropriate and adjusted adequately for the target group) | | 1 | | 3 | | 8 | 1 | 4 | | | 3 | | | | 5 | | | | 8 | | | | | 4 | | | | |  | | | 10 | | | 2 | | Morris 2014; Broekhuizen, 2012; Hamel 2013; Ogilvie 2007; Latimer 2010; Bock 2013; Carroll 2011; Ajie 2014; Brown 2012; Aalbers 2011; Salmon 2007; Heath 2006 |
|  | | 2 | | 1 | |  | 1 | 1 | | |  | | | | 1 | | | | 1 | | | | | 1 | | | | |  | | | 2 | | |  | | Nawaz 2001; Dixey 1999 |
|  | | 3 | |  | | 2 |  | 1 | | |  | | | | 1 | | | | 2 | | | | |  | | | | |  | | | 2 | | |  | | Cress 2006; |
| Writing up policies (defining priorities, objectives, draft, revisions) | | 2 | | 2 | |  |  | 1 | | | 1 | | | |  | | | | 1 | | | | |  | | | | | 1 | | | 2 | | |  | | Simovska2010; Netherlands Centre Youth Health 2013 |
| Plan for the program management | | 2 | | 1 | |  | 1 | 2 | | |  | | | |  | | | |  | | | | | 2 | | | | |  | | | 2 | | |  | | WHO 2011; Dixey 1999 |
| Manuals/exact protocols exist: exact description of content/components | | 1 | | 1 | |  |  |  | | |  | | | | 1 | | | | 1 | | | | |  | | | | |  | | | 1 | | |  | | Ghisi 2014 |
|  | | 2 | | 2 | | 1 | 1 | 4 | | |  | | | |  | | | | 2 | | | | | 2 | | | | |  | | | 4 | | |  | | Dixey 1999; Dubois 2008; WHO 2013; Netherlands Centre Youth Health 2013 |
| Common framework but flexible protocols (allowing for transfer to other countries) | | 3 | | 1 | |  |  |  | | | 1 | | | |  | | | |  | | | | | 1 | | | | |  | | | 1 | | |  | | Summerbell 2012 |
| The use of specific behavior change techniques: self-monitoring and self-management | | 1 | | 5 | |  | 1 |  | | |  | | | | 6 | | | | 2 | | | | | 4 | | | | |  | | | 5 | | | 1 | | Aldcroft 2011; Sumlin 2012; Tierney 2012; Michie 2009; Pearson 2012; Besculides 2008 |
| *Multidimensionality* | |  | |  | |  |  |  | | |  | | | |  | | | |  | | | | |  | | | | |  | | |  | | |  | |  |
| Multidimensionality of the approach (e.g., addressing individual, social, physical environment) | | 1 | | 3 | | 4 |  | 1 | | | 3 | | | | 3 | | | | 5 | | | | | 2 | | | | |  | | | 7 | | |  | | Flynn 2006; Walton-Moss 2013; Schröer 2014; Carroll 2011; Heath 2012; Salmon 2007; Kriemler 2011 |
|  | | 2 | | 7 | | 1 | 2 | 2 | | | 7 | | | | 1 | | | | 7 | | | | | 1 | | | | | 2 | | | 10 | | |  | | Trudnak 2012; ADA 2006; ADA 1999; Haughton 2012; Pratt 2008; Hoelscher 2013; WHO 2011; Simovska 2010; Dixey 1999 |
|  | | 3 | |  | | 1 | 1 | 2 | | |  | | | |  | | | | 1 | | | | |  | | | | | 1 | | | 2 | | |  | | Bellew 2008; Swinburn 2005 Foltz 2012 |
| Collaborative approach: ensuring commitment of the managers/staff at the locations for policy, consensus with community | | 2 | | 1 | |  |  |  | | | 1 | | | |  | | | |  | | | | |  | | | | | 1 | | | 1 | | |  | | Simovska 2010 |
| Accounts for physical environment (environmental structures, transportation, land use) | | 1 | | 2 | | 2 |  | 1 | | | 3 | | | |  | | | | 2 | | | | | 2 | | | | |  | | | 4 | | |  | | Ayliffe 2010; Biddle 2014; Hendrie 2012; Heath 2012 |
|  | | 2 | | 1 | |  |  |  | | | 1 | | | |  | | | | 1 | | | | |  | | | | |  | | | 1 | | |  | | ADA 2006 |
|  | | 3 | |  | | 1 | 1 | 2 | | |  | | | |  | | | |  | | | | |  | | | | | 2 | | | 2 | | |  | | Brennan Ramirez 2006; Bellew 2008 |
| Multidisciplinary development of policy/intervention | | 1 | | 1 | |  |  |  | | | 1 | | | |  | | | | 1 | | | | |  | | | | |  | | | 1 | | |  | | Ayliffe 2010 |
| *Practitioner and setting context* | |  | |  | |  |  |  | | |  | | | |  | | | |  | | | | |  | | | | |  | | |  | | |  | |  |
| Practitioners' skills, training, and individual characteristics | | 1 | | 7 | | 1 | 2 |  | | | 2 | | | | 8 | | | | 5 | | | | | 5 | | | | |  | | | 9 | | | 1 | | Greaves 2011; Burrows 2012; Acery 2013; van Achterberg 2011; Paul-Ebhohimhen 2009; Ghisi 2014; Flynn 2006; Besculides 2008; Vuillemin 2011 |
|  | | 2 | |  | |  | 1 |  | | |  | | | | 1 | | | | 1 | | | | |  | | | | |  | | | 1 | | |  | | Haughton 2012 |
|  | | 3 | | 2 | |  |  | 1 | | | 1 | | | |  | | | | 2 | | | | |  | | | | |  | | | 2 | | |  | | Gillison 2012; Weaver 2012 |
|  | |  | |  | |  |  |  | | |  | | | |  | | | |  | | | | |  | | | | |  | | |  | | |  | |  |
| Stakeholder as a partner in the development of policy | | 1 | | 1 | |  |  |  | | | 1 | | | |  | | | | 1 | | | | |  | | | | |  | | | 1 | | |  | | Richardson 2013 |
|  | | 2 | | 1 | | 1 |  | 2 | | |  | | | |  | | | |  | | | | | 2 | | | | |  | | | 2 | | |  | | WHO 2011; WHO 2013 |
|  | | 3 | | 2 | | 1 | 1 | 4 | | |  | | | |  | | | | 1 | | | | |  | | | | | 3 | | | 4 | | |  | | Christiansen 2014; Bellew 2008; Swinburn 2005; King 2011 |
| Setting and its characteristics well defined | | 1 | | 9 | | 8 | 2 | 1 | | | 11 | | | | 7 | | | | 11 | | | | | 8 | | | | |  | | | 15 | | | 4 | | Rabin 2010; Dombrowski 2012; Greaves 2011; Avery 2013; Ayliffe 2010; Biddle 2014; Hendrie 2012; van Achterberg 2011; de Meester 2009; Kriemler 2011; Ajie 2014; Ghisi 2014; Hamel 2013; Campbell 2007; Marsh 2014; van Sluijs 2008; Salmon 2007; Stephens 2014; Vuillemin 2011; |
|  | | 2 | | 2 | | 1 | 1 | 2 | | | 2 | | | |  | | | | 4 | | | | |  | | | | |  | | | 4 | | |  | | Stockley 2001; ADA 1999; Dubois 2008 |
|  | | 3 | | 2 | |  |  | 1 | | | 1 | | | |  | | | | 2 | | | | |  | | | | |  | | | 2 | | |  | | Weaver 2012; Glasgow 2007; Foltz 2012 |
| Barriers specific for the setting; access to the setting | | 2 | | 1 | |  | 1 |  | | | 2 | | | |  | | | | 2 | | | | |  | | | | |  | | | 2 | | |  | | Trudnak 2012; ADA 1999 |
| Infrastructure development addressed | | 3 | |  | | 1 |  | 1 | | |  | | | |  | | | |  | | | | |  | | | | | 1 | | | 1 | | |  | | Christiansen 2014 |
| Marketing in settings | | 2 | | 1 | |  |  |  | | | 1 | | | |  | | | | 1 | | | | |  | | | | |  | | | 1 | | |  | | ADA 2003 |
| Fitting into existing policies/actions | | 2 | | 1 | |  | 1 | 1 | | | 1 | | | |  | | | |  | | | | | 1 | | | | | 1 | | | 2 | | |  | | Simovska 2010;  Dixey 1999 |
|  | | 3 | | 1 | |  |  | 1 | | |  | | | |  | | | |  | | | | |  | | | | | 1 | | | 1 | | |  | | Swinburn 2005 |
| *Efficiency established* | | |  |  | |  |  |  | |  | | | |  | | | | | | | |  | | | |  | |  | | | | |  | |  | |  |
| Efficiency: significant effects established in prior trials | | |  |  | |  |  |  | |  | | | |  | | | | | | | |  | | | |  | |  | | | | |  | |  | | All systematic reviews, all stakeholders documents |
|  | | | 3 | - | | 1 | - | 1 | | - | | | | - | | | | | | | | 1 | | | | - | | - | | | | | 1 | | - | | Heath 2006 |
| *Costs and funding* | | |  |  | |  |  |  | |  | | | |  | | | | | | | |  | | | |  | |  | | | | |  | |  | |  |
| Costs related to health benefits (including population health change, morbidity, mortality, QOL) | | | 1 | 1 | | 2 | - | 1 | | - | | | | 2 | | | | | | | | 3 | | | | - | | - | | | | | 3 | | - | | Hoehner 2013; Roux 2008; Anderson 2009 |
|  | | | 2 | 2 | |  | 1 | 2 | | 1 | | | |  | | | | | | | | 2 | | | | 1 | |  | | | | | 3 | |  | | Stockley 2001; Pratt 2008; Weightman 2005 |
| Costs related to behavior change (e.g. hours of PA gained per person) | | | 1 | 3 | | 3 |  | 4 | |  | | | | 2 | | | | | | | | 5 | | | | 1 | |  | | | | | 4 | | 2 | | Hoehner 2013; Wu 2011; Rabin 2010; Anderson 2009; Brown 2012; Goode 2012 Heath 2006 |
|  | | | 2 | 2 | |  | 1 | 2 | | 1 | | | |  | | | | | | | | 2 | | | | 1 | |  | | | | | 3 | |  | | Stockley 2001; Pratt 2008; Weightman 2005 |
|  | | | 3 | 1 | | 1 |  | 2 | |  | | | |  | | | | | | | | 2 | | | |  | |  | | | | | 2 | |  | | Katz 2005 |
| Monitoring of funds and human resources | | | 2 | 1 | |  |  | 1 | |  | | | |  | | | | | | | |  | | | | 1 | |  | | | | | 1 | |  | | WHO 2011 |
| Economic improvement/development strategies | | | 1 |  | | 1 |  | 1 | |  | | | |  | | | | | | | | 1 | | | |  | |  | | | | | 1 | |  | | Heath 2006 |
| Total costs of the interventions/policy (total budget per participant) | | | 1 | 4 | | 3 |  | 4 | | 2 | | | | 1 | | | | | | | | 6 | | | | 1 | |  | | | | | 6 | | 1 | | Hoehner 2013; Wu 2011; Klesges 2012; Anderson 2009; Ogilvie 2007; Klesges 2008; Goode 2012 |
|  | | | 2 | 1 | |  |  | 1 | |  | | | |  | | | | | | | | 1 | | | |  | |  | | | | | 1 | |  | | Netherlands Centre Youth Health 2013 |
|  | | | 3 | 2 | | 2 |  | 4 | |  | | | |  | | | | | | | | 4 | | | |  | |  | | | | | 4 | |  | | Christiansen 2014; Kahn 2002; Glasgow 2007; McNeil 2006; |
| *Outcomes* | | |  |  | |  |  |  | |  | | | |  | | | | | | | |  | | | |  | |  | | | | |  | |  | |  |
| Outcomes measured with valid, reliable, and sensitive tools | | |  |  | |  |  |  | |  | | | |  | | | | | | | |  | | | |  | |  | | | | |  | |  | | All systematic reviews; all stakeholders documents |
| Effects specified as clinically significant (symptom reduction, moving from sedentary to physically active, obesity reduction, QOL) | | | 1 | 4 | | 2 |  | 1 | | 3 | | | | 2 | | | | | | | | 5 | | | | 1 | |  | | | | | 6 | |  | | Roux 2008; Klesges 2012; Taylor 2007; Lombard 2009; Katz 2008; Klesges 2008; |
|  | | | 2 | 3 | |  |  | 3 | | 1 | | | |  | | | | | | | | 2 | | | | 1 | |  | | | | | 3 | |  | | Khan 2009; Netherlands Centre Youth Health 2013; WHO 2011 |
|  | | | 3 | 3 | |  |  | 2 | | 2 | | | |  | | | | | | | | 3 | | | |  | |  | | | | | 3 | |  | | Gillison 2012; Katz 2005; Weaver 2012 |
| Effects on public health-relevant secondary outcomes (proximal, such as weight loss and distal, such as heart disease) | | | 1 | 3 | | 2 |  |  | | 1 | | | | 4 | | | | | | | | 4 | | | | 1 | |  | | | | | 5 | |  | | Schröer 2014; Marsh 2014; Anderson 2009; Kirk 2012; Vuillemin 2011 |
|  | | | 2 | 1 | |  |  | 1 | |  | | | |  | | | | | | | |  | | | | 1 | |  | | | | | 1 | |  | | NCI 2012 |
|  | | | 2 | 4 | |  | 2 | 5 | |  | | | |  | | | | | | | | 3 | | | | 3 | |  | | | | | 5 | |  | | Stockley 2001; Spencer 2013; Netherlands Centre Youth Health 2013; NCI 2012; WHO 2011 |
| Program results evaluated as useful by participants stakeholders, program providers, and funding agencies | | | 1 | 1 | |  |  |  | | 1 | | | |  | | | | | | | | 1 | | | |  | |  | | | | | 1 | |  | | Flynn 2006 |
| Evaluation by stakeholders included | | | 2 | 1 | |  |  | 1 | |  | | | |  | | | | | | | |  | | | | 1 | |  | | | | | 1 | |  | | WHO 2011 |
| Benefits/risk ratio | | | 2 | 1 | |  |  | 1 | |  | | | |  | | | | | | | | 1 | | | |  | |  | | | | | 1 | |  | | Spencer 2013 |
| Confounding factors for the effects addressed | | | 2 | 1 | |  |  | 1 | |  | | | |  | | | | | | | |  | | | | 1 | |  | | | | | 1 | |  | | WHO 2011 |
| Environmental and social benefits | | | 1 |  | | 1 |  | 1 | |  | | | |  | | | | | | | | 1 | | | |  | |  | | | | | 1 | |  | | Hoehner 2013 |
| Ability of the intervention/policy to achieve health equity | | | 2 | 1 | |  |  | 1 | |  | | | |  | | | | | | | | 1 | | | |  | |  | | | | | 1 | |  | | Spencer 2013 |
| Risks/negative consequences evaluated | | | 1 | 4 | | 2 |  | 1 | | 2 | | | | 3 | | | | | | | | 5 | | | | 1 | |  | | | | | 6 | |  | | Anderson 2009; Klesges 2008; Gudzune 2013; Ogilvie 2007; Brennan 2014; Vuillemin 2011 |
|  | | | 2 | 4 | |  |  | 3 | |  | | | | 1 | | | | | | | | 3 | | | | 1 | |  | | | | | 4 | |  | | Nawaz 2001; WHO 2011; Spencer 2013; Netherlands Centre Youth Health 2013; |
|  | | | 3 | 1 | | 1 | 1 | 2 | |  | | | | 1 | | | | | | | | 3 | | | |  | |  | | | | | 3 | |  | | Cress 2006; Kahn 2002; Glasgow 2007 |
| Measured outcomes include physiological risk factor indices (e.g., BMI, cholesterol) | | | 1 | 4 | |  | 1 |  | | 2 | | | | 3 | | | | | | | | 2 | | | | 3 | |  | | | | | 4 | | 1 | | Aldcroft 2011; Broekhuizen 2012; Hamel 2013; Kriemler 2011; De Bourdeaudhuij 2011 |
| Outcomes measured with valid, reliable, and sensitive tools | | | 2 | 2 | | 1 |  | 3 | |  | | | |  | | | | | | | | 1 | | | | 2 | |  | | | | | 3 | |  | | WHO 2011; Dixey 1999; Dubois 2008 |
| *Effects’ evaluation: time and effect size* | | | | | |  |  |  | |  | | | |  | | | | | | | |  | | | |  | |  | | | | |  | |  | |  |
| Sustainable effects: mid-term effects (+6 months) or long term effects (+12 months) | | | 1 | 8 | | 3 | 1 | 2 | | 3 | | | | 7 | | | | | | | | 4 | | | | 8 | |  | | | | | 9 | | 3 | | Aldcroft 2011; Klesges 2012; Kohl 2013; Broekhuizen 2012; Geaney 2013; Paul-Ebhohimhen 2009; Marsh 2014; Brennan 2014; Müller-Riemenschneider 2008; Gudzune 2013; Goode 2012; Vuillemin 2011 |
|  | | | 2 | 4 | |  |  | 3 | | 1 | | | | 1 | | | | | | | | 3 | | | | 2 | |  | | | | | 5 | |  | | Khan 2009; Nawaz 2001; NCI 2012; WHO 2011; Spencer 2013 |
|  | | | 3 | 2 | | 1 |  | 3 | |  | | | |  | | | | | | | | 3 | | | |  | |  | | | | | 3 | | 1 | | Bellew 2008; Glasgow 2007; Gillison 2012 |
| Effect sizes (besides significant effects) | | | 1 | 3 | | 4 | 1 |  | |  | | | | 8 | | | | | | | | 3 | | | | 5 | |  | | | | | 5 | | 3 | | Bossen 2014; Robertson 2012; Lee 2010; Geraedts 2013; Dombrowski 2012; Greaves 2011; Broekhuizen 2012; Geaney 2013 |
|  | | | 2 | 2 | | 2 |  | 3 | |  | | | |  | | | | | | | | 2 | | | | 1 | | 1 | | | | | 4 | |  | | Dubois 2008; WHO 2013; WHO 2013 (separate for intervention and policies; Netherlands Centre Youth Health 2013 |
|  | | | 3 | 1 | | 1 |  | 2 | |  | | | |  | | | | | | | | 2 | | | |  | |  | | | | | 2 | |  | | Christiansen 2014; Swinburn 2005 |
| Specific plans for evaluation | | | 2 |  | | 1 |  | 1 | |  | | | |  | | | | | | | |  | | | | 1 | |  | | | | | 1 | |  | | Dixey 1999 |
| Procedures of evaluation communicated to all responsible for the intervention | | | 3 |  | | 1 |  | 1 | |  | | | |  | | | | | | | | 1 | | | |  | |  | | | | | 1 | |  | | Christiansen 2014 |
| Evaluation of implementation procedures included | | | 3 |  | | 1 |  | 1 | |  | | | |  | | | | | | | | 1 | | | |  | |  | | | | |  | | 1 | | Bellew 2008 |
| *Reach* | | |  |  | |  |  |  | |  | | | |  | | | | | | | |  | | | |  | |  | | | | |  | |  | |  |
| Reach (the strategy is likely to involve a large percentage of the target population; reaching entire target population) | | | 1 | 4 | | 3 |  | 1 | | 3 | | | | 3 | | | | | | | | 5 | | | | 2 | |  | | | | | 6 | | 1 | | Hoehner 2013; Wilding 2013; Klesges 2012; Kohl 2013; Brennan 2014; Vuillemin 2011; Klesges 2008 Heath 2006 |
|  | | | 2 | 4 | | 1 | 1 | 4 | | 1 | | | | 1 | | | | | | | | 5 | | | |  | | 1 | | | | | 6 | |  | | Khan 2009; Haughton 2012; Dubois 2008; WHO 2013; NCI 2012; Spencer 2013 |
|  | | | 3 | 3 | | 1 | 1 | 4 | |  | | | | 1 | | | | | | | | 4 | | | |  | | 1 | | | | | 5 | |  | | Besculides 2008; Bellew 2008; Glasgow 2007; McNeil 2006; |
| Inclusiveness in terms of individuals with low mobility or with comorbidities; accounting for risk and susceptibility factors, including high risk individuals, different age and gender groups | | | 1 | 4 | | 3 |  | 1 | | 3 | | | | 3 | | | | | | | | 5 | | | | 2 | |  | | | | | 6 | | 1 | | Hoehner 2013; Wilding 2013; Klesges 2012; Kohl 2013; Brennan 2014; Vuillemin 2011; Klesges 2008 Heath 2006 |
|  | | | 2 | 6 | | 2 | 1 | 7 | |  | | | | 2 | | | | | | | | 5 | | | | 3 | | 1 | | | | | 9 | |  | | Stockley 2001; Nawaz 2001; WHO 2011; Dixey 1999; Dubois 2008; Netherlands Centre Youth Health 2013; WHO 2013; WHO 2013 (separate entries for intervention and policies) |
|  | | | 3 | 1 | | 1 |  | 2 | |  | | | |  | | | | | | | | 2 | | | |  | |  | | | | | 2 | |  | | Gillison 2012; Foltz 2012 |
| Cultural competence/social inclusiveness of intervention/policy: accounts for cultural/minority issues in recruitment processes, content, setting) | | | 1 | 5 | | 5 | 2 | 3 | | 4 | | | | 5 | | | | | | | | 8 | | | | 4 | |  | | | | | 12 | |  | | Flynn 2006; Sumlin 2012; Brennan 2014; Bock 203; Thomson 2011; Ogilvie 2007; Vuillemin 2011; Hoehner 2013; Wilding 2013; Klesges 2012; Kohl 2013; Klesges 2008 |
|  | | | 2 | 4 | | 1 | 1 | 3 | | 2 | | | | 1 | | | | | | | | 5 | | | |  | | 1 | | | | | 6 | |  | | Haughton 2012; Pratt 2008; Dubois 2008; IMPALA 2011; Spencer 2013; ADA 2003 |
|  | | | 3 | 2 | |  |  | 1 | | 1 | | | |  | | | | | | | | 1 | | | | 1 | |  | | | | | 2 | |  | | Summerbell 2012; King 2011 |
| Reach evaluated with qualitative methods | | | 2 | 1 | |  |  | 1 | |  | | | |  | | | | | | | |  | | | | 1 | |  | | | | | 1 | |  | | NCI 2012 |
| *Participation and generalizability of evaluation* | | |  |  | |  |  |  | |  | | | |  | | | | | | | |  | | | |  | |  | | | | |  | |  | |  |
| Generalizability of effects evaluated (evaluations if effects observed across different types of participants, at the population level) | | | 1 | 3 | | 1 |  |  | | 4 | | | |  | | | | | | | | 4 | | | |  | |  | | | | | 4 | |  | | Klesges 2012; Katz 2008; Kriemler 2011; Brennan 2014; |
|  | | | 2 | 2 | |  |  | 2 | |  | | | |  | | | | | | | | 1 | | | | 1 | |  | | | | | 2 | |  | | Spencer 2013; NCI 2012 |
| Evaluation of effectiveness across settings | | | 1 | 2 | | 1 |  |  | | 2 | | | | 1 | | | | | | | | 2 | | | | 1 | |  | | | | | 3 | |  | | Dombrowski 2012; Campbell 2007; Marsh 2014 |
| The proportion of individuals affected, compared to those who could be affected by the action | | | 2 | 1 | |  |  | 1 | |  | | | |  | | | | | | | | 1 | | | |  | |  | | | | | 1 | |  | | Spencer 2013 |
| Change related to the objective | | | 2 | 1 | |  |  | 1 | |  | | | |  | | | | | | | | 1 | | | |  | |  | | | | | 1 | |  | | Netherlands Centre Youth Health 2013 |
| Effects in priority populations (due to health risks) evaluated | | | 1 | 1 | |  |  |  | | 1 | | | |  | | | | | | | | 1 | | | |  | |  | | | | | 1 | |  | | Brennan 2014 |
|  | | | 3 | 1 | |  |  | 1 | |  | | | |  | | | | | | | | 1 | | | |  | |  | | | | | 1 | |  | | Swinburn 2005 |
| Participation rates | | | 1 | 5 | |  |  | 1 | | 1 | | | | 3 | | | | | | | | 5 | | | |  | |  | | | | | 5 | |  | | Klesges 2012; Rabin 2010; Lombard 2009; Klesges 2008; Gudzune 2013; |
|  | | | 3 | 1 | |  |  | 1 | |  | | | |  | | | | | | | | 1 | | | |  | |  | | | | | 1 | |  | | Glasgow 2007 |
|  | | | 2 | 4 | |  |  | 4 | |  | | | |  | | | | | | | | 2 | | | | 2 | |  | | | | | 4 | |  | | WHO 2011; Dubois 2008; Spencer 2013; NCI 2012 |
| *Processes and active components* | | |  |  | |  |  |  | |  | | | |  | | | | | | | |  | | | |  | |  | | | | |  | |  | |  |
| Active components identified | | | 1 | 5 | |  |  |  | | 1 | | | | 4 | | | | | | | | 2 | | | | 3 | |  | | | | | 2 | | 3 | | Dombrowski 2012; Michie 2009; Prestwich 2014; Anderson 2009; Flynn 2006 |
|  | | | 3 | 1 | |  |  | 1 | |  | | | |  | | | | | | | | 1 | | | |  | |  | | | | | 1 | |  | | Swinburn 2005 |
| Accuracy standards and accuracy evaluation (evaluations provides information on quality of delivery/features that determine the worth of the program) | | | 1 | 2 | |  |  |  | | 1 | | | | 1 | | | | | | | | 1 | | | | 1 | |  | | | | | 1 | | 1 | | Michie 2009; Flynn 2006 |
|  | | | 2 | 1 | |  | 1 | 2 | |  | | | |  | | | | | | | | 1 | | | | 1 | |  | | | | | 2 | |  | | Stockley 2001; WHO 2011 |
| Intervention process measured/policy surveillance (what, when, where delivered and how assessed) | | | 1 | 1 | |  |  | 1 | |  | | | |  | | | | | | | | 1 | | | |  | |  | | | | | 1 | |  | | Lombard 2009 |
|  | | | 2 | 1 | | 1 |  | 2 | |  | | | |  | | | | | | | |  | | | | 1 | | 1 | | | | | 2 | |  | | WHO 2011; WHO 2013 |
| Ongoing monitoring/measurement of delivery according to protocol; monitoring of materials | | | 2 | 2 | | 1 |  | 3 | |  | | | |  | | | | | | | |  | | | | 2 | | 1 | | | | | 3 | |  | | Simovska 2010; WHO 2013 ; WHO 2011; Stockley, 2001 |
|  | | | 1 | 2 | | 1 |  | 1 | |  | | | | 2 | | | | | | | | 1 | | | | 2 | |  | | | | | 3 | |  | | Gudzune 2013; Vuillemin 2011 Lombard 2009 |
| *Participation processes* | | |  |  | |  |  |  | |  | | | |  | | | | | | | |  | | | |  | |  | | | | |  | |  | |  |
| Completion and attrition rates across the stages of the program | | | 1 | 3 | | 2 |  |  | | 1 | | | | 4 | | | | | | | | 2 | | | | 3 | |  | | | | | 4 | | 1 | | Robertson 2012; Klesges 2012; Kohl 2013; Aalbers 2011; Vuillemin 2011 |
|  | | | 2 | 2 | |  |  | 2 | |  | | | |  | | | | | | | |  | | | | 2 | |  | | | | | 2 | |  | | WHO 2011; NCI 2012 |
|  | | | 3 | 1 | |  |  | 1 | |  | | | |  | | | | | | | | 1 | | | |  | |  | | | | | 1 | |  | | Glasgow 2007 |
| Resources and strategies for practitioners helping them to invite and follow participants up | | | 1 | 2 | | 2 |  | 1 | | 2 | | | | 1 | | | | | | | | 3 | | | | 1 | |  | | | | | 4 | |  | | Katz 2008; Heath 2012; Hearn 2008; Carroll 011 |
|  | | | 2 | 1 | | 1 |  | 1 | |  | | | | 1 | | | | | | | | 1 | | | | 1 | |  | | | | | 2 | |  | | Nawaz 2001; WHO 2013 |
| Recruitment enhanced by partnering with stakeholders | | | 1 |  | | 1 |  |  | |  | | | | 1 | | | | | | | | 1 | | | |  | |  | | | | | 1 | |  | | Carroll, 2011 |
| Retention enhanced by partnering community and stakeholders | | | 1 |  | | 1 |  |  | |  | | | | 1 | | | | | | | | 1 | | | |  | |  | | | | | 1 | |  | | Carroll, 2011 |
| Proactive referral | | | 3 | 1 | |  |  |  | |  | | | | 1 | | | | | | | | 1 | | | |  | |  | | | | | 1 | |  | | Besculides 2008 |
| Tracking system identifying those in need | | | 3 | 1 | |  |  |  | |  | | | | 1 | | | | | | | | 1 | | | |  | |  | | | | | 1 | |  | | Besculides 2008 |
|  | | |  |  | |  |  |  | |  | | | |  | | | | | | | |  | | | |  | |  | | | | |  | |  | |  |
| Barriers referring to stigmatization measured and prevented | | | 3 | 1 | |  |  | 1 | |  | | | |  | | | | | | | | 1 | | | |  | |  | | | | | 1 | |  | | Katz 2005 |
| Recruitment from community-based settings to increase participation | | | 1 |  | | 1 |  |  | |  | | | | 1 | | | | | | | | 1 | | | |  | |  | | | | | 1 | |  | | Wilding 2013 |
| Strategies to increase access to intervention | | | 1 | 1 | |  |  |  | | 1 | | | |  | | | | | | | | 1 | | | |  | |  | | | | | 1 | |  | | Ayliffe 2010 |
| Incentives for participation and participation engagement techniques | | | 3 | 3 | |  |  |  | | 1 | | | | 2 | | | | | | | | 2 | | | |  | |  | | | | | 3 | |  | | Weaver 2012; Besculides 2008; King 2011 |
|  | | | 2 | 1 | |  |  |  | |  | | | | 1 | | | | | | | | 1 | | | |  | |  | | | | | 1 | |  | | Nawaz 2001 |
| Active participation of target population achieved | | | 2 | 3 | |  |  | 2 | |  | | | | 1 | | | | | | | |  | | | | 3 | |  | | | | | 3 | |  | | Dixey 1999, WHO 2013, Nawaz, 2001 |
| Strategies promoting long-term participation (maintenance) included | | | 1 | 3 | | 2 | 1 | 1 | | 1 | | | | 4 | | | | | | | | 4 | | | | 2 | |  | | | | | 6 | |  | | Morris 2014; Ayliffe 2010; Carroll 2011; Müller-Riemenschneider 2008; Besculides 2008; Gillison 2012 |
| *Training for practitioners* | | |  |  | |  |  |  | |  | | | |  | | | | | | | |  | | | |  | |  | | | | |  | |  | |  |
| Training for staff in implementation and facilitating inter-sectorial collaboration | | | 1 | 2 | | 2 |  | 2 | | 1 | | | | 1 | | | | | | | | 2 | | | | 2 | |  | | | | | 3 | | 1 | | Heath 2012; Klesges 2012; Goode 2012; Vuillemin 2011 |
|  | | | 3 | 1 | |  |  | 1 | |  | | | |  | | | | | | | | 1 | | | |  | |  | | | | | 1 | |  | | Glasgow 2007 |
| Developing goals for community and staff | | | 1 | 1 | |  |  |  | | 1 | | | |  | | | | | | | | 1 | | | |  | |  | | | | | 1 | |  | | Katz 2008 |
| Managers trained, their roles clarified | | | 2 |  | | 1 |  | 1 | |  | | | |  | | | | | | | |  | | | | 1 | |  | | | | | 1 | |  | | Dixey 1999 |
| Necessary expertise for implementation specified | | | 2 | 1 | |  |  | 1 | |  | | | |  | | | | | | | | 1 | | | |  | |  | | | | | 1 | |  | | Spencer 2013 |
| *Use/integration of existing resources* | | |  |  | |  |  |  | |  | | | |  | | | | | | | |  | | | |  | |  | | | | |  | |  | |  |
| Implementation integrated into existing programs | | | 1 |  | | 1 |  | 1 | |  | | | |  | | | | | | | | 1 | | | |  | |  | | | | | 1 | |  | | Hoehner 2013 |
|  | | | 2 | 4 | |  |  | 3 | | 1 | | | |  | | | | | | | | 1 | | | | 2 | | 1 | | | | | 4 | |  | | Simovska 2010; WHO 2011; Spencer 2013; NCI 2012 |
| Ongoing support from stakeholders secured | | | 2 | 4 | |  |  | 3 | | 1 | | | |  | | | | | | | | 2 | | | | 1 | | 1 | | | | | 4 | |  | | Turndak 2012; IMPALA 2011; WHO 2011; Netherlands Centre Youth Health 2013 |
|  | | | 3 | 1 | |  |  | 1 | |  | | | |  | | | | | | | | 1 | | | |  | |  | | | | | 1 | |  | | Gillison 2012 |
| Tools helping to put recommendations into practice | | | 2 | 1 | |  |  |  | | 1 | | | |  | | | | | | | | 1 | | | |  | |  | | | | | 1 | |  | | ADA 1999 |
| Support for family engagement | | | 2 | 1 | |  |  | 1 | |  | | | |  | | | | | | | | 1 | | | |  | |  | | | | | 1 | |  | | Hoelscher 2013 |
| Accounting for location of practitioners/ geographical access to practitioners | | | 2 |  | |  | 1 |  | |  | | | | 1 | | | | | | | | 1 | | | |  | |  | | | | | 1 | |  | | Haughton 2012 |
| Adaptation to local circumstances/existing infrastructure | | | 1 |  | | 1 |  |  | |  | | | | 1 | | | | | | | |  | | | | 1 | |  | | | | | 1 | |  | | Vuillemin 2011 |
|  | | | 2 | 1 | |  |  |  | | 1 | | | |  | | | | | | | | 1 | | | |  | |  | | | | | 1 | |  | | Pratt 2008 |
| Setting adjusted to the program | | | 1 | 1 | |  |  |  | | 1 | | | |  | | | | | | | | 1 | | | |  | |  | | | | | 1 | |  | | Klesges 2012 |
| Setting: Access, safety, ecological standards, design | | | 2 |  | | 1 |  | 1 | |  | | | |  | | | | | | | |  | | | |  | | 1 | | | | | 1 | |  | | IMPALA 2011 |
| Public acceptance for financing | | | 2 |  | | 1 |  | 1 | |  | | | |  | | | | | | | |  | | | |  | | 1 | | | | | 1 | |  | | IMPALA 2011 |
| Financing infrastructures, their maintenance, lifecycle costs | | | 2 |  | | 1 |  | 1 | |  | | | |  | | | | | | | |  | | | |  | | 1 | | | | | 1 | |  | | IMPALA 2011 |
| Financial consultations | | | 2 |  | | 1 |  | 1 | |  | | | |  | | | | | | | |  | | | |  | | 1 | | | | | 1 | |  | | IMPALA 2011 |
| Levels of upstream investments involved in the program | | | 1 | 1 | |  |  |  | | 1 | | | |  | | | | | | | | 1 | | | |  | |  | | | | | 1 | |  | | Flynn 2006 |
| Resources invested and obtained during implementation | | | 2 | 1 | |  |  | 1 | |  | | | |  | | | | | | | |  | | | | 1 | |  | | | | | 1 | |  | | WHO 2011 |
| Resources for implementation specified | | | 2 | 1 | | 2 |  | 3 | |  | | | |  | | | | | | | | 1 | | | | 1 | | 1 | | | | | 3 | |  | | Dixey 1999; Spencer 2013; WHO 2013; |
| Resources for materials planned | | | 2 |  | |  | 1 | 1 | |  | | | |  | | | | | | | |  | | | | 1 | |  | | | | | 1 | |  | | Dixey 1999 |
| Plans for resources, schedule, and infrastructure | | | 2 |  | | 1 | 1 | 2 | |  | | | |  | | | | | | | |  | | | | 1 | | 1 | | | | | 2 | |  | | Dixey 1999; IMPALA 2011 |
| Barriers and resources for implementation | | | 2 | 1 | | 1 |  | 1 | | 1 | | | |  | | | | | | | | 2 | | | |  | |  | | | | | 2 | |  | | ADA 1999; Spencer 2013 |
| Time allocation plans | | | 2 |  | | 1 |  | 1 | |  | | | |  | | | | | | | |  | | | | 1 | |  | | | | | 1 | |  | | Dixey 1999 |
| *Feasibility* | | |  |  | |  |  |  | |  | | | |  | | | | | | | |  | | | |  | |  | | | | |  | |  | |  |
| Propriety (standards ensuring that the program was fitting ethical and legal standards) | | | 2 |  | |  | 1 | 1 | |  | | | |  | | | | | | | |  | | | | 1 | |  | | | | | 1 | |  | | Dixey 1999 |
|  | | | 1 | 1 | |  |  |  | | 1 | | | |  | | | | | | | | 1 | | | |  | |  | | | | | 1 | |  | | Flynn 2006 |
| Adoption by target staff, settings, or institutions (representativeness, exclusion, characteristics of those who adopted vs those who did not) | | | 1 | 4 | |  |  |  | | 2 | | | | 2 | | | | | | | | 3 | | | | 1 | |  | | | | | 3 | | 1 | | Rabin 2010; Brennan 2014; Klesges 2008; Kohl 2013 |
|  | | | 2 | 2 | |  |  | 2 | |  | | | |  | | | | | | | | 1 | | | | 1 | |  | | | | | 2 | |  | | Dubois 2008; NCI 2012 |
| Feasible/acceptable for providers (fitting their skills; no external specialists need), stakeholders, and participants | | | 1 | 4 | | - | - | 1 | | 2 | | | | 1 | | | | | | | | 4 | | | | - | | - | | | | | 4 | | - | | Flynn 2006; Brennan 2014; Hoehner 2013; Rabin, 2010 |
|  | | | 3 | 4 | | 1 |  | 4 | |  | | | | 1 | | | | | | | | 4 | | | |  | | 1 | | | | | 5 | |  | | Gillison 2012;  Swinburn 2005; McNeil 2006; Kahn 2002; Besculides 2008 |
| Attitudes of providers facilitating implementation | | | 1 | 1 | |  |  |  | | 1 | | | |  | | | | | | | | 1 | | | |  | |  | | | | | 1 | |  | | Katz 2008 |
| *Maintenance-sustainability* | | |  |  | |  |  |  | |  | | | |  | | | | | | | |  | | | |  | |  | | | | |  | |  | |  |
| Maintenance (maintained with institutional support); continuation within the realm of the institution | | | 1 | 3 | | 1 |  | 1 | | 2 | | | | 1 | | | | | | | | 4 | | | |  | |  | | | | | 4 | |  | | Hoehner 2013; Rabin 2010 Brennan 2014; Klesges 2008 |
|  | | | 2 | 1 | | 1 |  | 2 | |  | | | |  | | | | | | | | 1 | | | | 1 | |  | | | | | 2 | |  | | Spencer 2013; Dixey 1999 |
| Program or its components institutionalized after completion | | | 1 |  | | 1 |  |  | |  | | | | 1 | | | | | | | |  | | | | 1 | |  | | | | | 1 | |  | | Vuillemin 2011 |
|  | | | 3 | 3 | |  |  | 3 | |  | | | |  | | | | | | | | 2 | | | |  | | 1 | | | | | 3 | |  | | Swinburn 2005; Glasgow 2007; McNeil 2007 |
| Mutability (intervention/policy is in the realm of the community) | | | 2 | 5 | |  |  | 4 | | 1 | | | |  | | | | | | | | 3 | | | | 2 | |  | | | | | 5 | |  | | Khan 2009; Dixey 1999; Dubois 2008; Spencer 2013; NCI 2012 |
|  | | | 3 | 3 | |  |  | 3 | |  | | | |  | | | | | | | | 1 | | | |  | | 1 | | | | | 3 | |  | | Swinburn 2005; Glasgow 2007; McNeil 2007 |
| Financial sustainability | | | 2 | 1 | |  |  | 1 | |  | | | |  | | | | | | | | 1 | | | |  | |  | | | | | 1 | |  | | Spencer 2013 |
| Sustaining training for program operations | | | 3 | 1 | |  |  |  | |  | | | | 1 | | | | | | | | 1 | | | |  | |  | | | | | 1 | |  | | Besculides 2008 |
| Adequate resources by stakeholders and sustainability of their investment | | | 3 | 1 | | 1 |  | 2 | |  | | | |  | | | | | | | | 2 | | | |  | |  | | | | | 1 | | 1 | | Bellew 2008; King 2011 |
| Community engagement strategies (analysis, planning, and information for community; community resources and its capacity to secure implementation and sustainability) | | | 3 | 1 | |  |  | 1 | |  | | | |  | | | | | | | | 1 | | | |  | |  | | | | | 1 | |  | | King 2011 |
| Evaluation of conditions related to sustainability of intervention | | | 1 |  | | 1 |  |  | |  | | | | 1 | | | | | | | |  | | | | 1 | |  | | | | | 1 | |  | | Vuillemin 2011 |
| *Partnership for implementation* | | |  |  | |  |  |  | |  | | | |  | | | | | | | |  | | | |  | |  | | | | |  | |  | |  |
| Partnership between agencies/organizations (e.g. school, business, transport agencies) to facilitate adoption and implementation – intersectorial collaboration of stakeholders | | | 1 |  | | 1 |  | 1 | |  | | | |  | | | | | | | |  | | | | 1 | |  | | | | | 1 | |  | | Heath 2012 |
|  | | | 2 | 4 | | 1 |  | 2 | | 3 | | | |  | | | | | | | | 3 | | | | 1 | | 1 | | | | | 5 | |  | | ADA 2003; IMPALA 2011; WHO 2011 Turndak 2012; Pratt 2008 |
| Collaboration between providers | | | 1 | 1 | |  |  |  | | 1 | | | |  | | | | | | | | 1 | | | |  | |  | | | | | 1 | |  | | Katz 2008 |
| Networks for implementation | | | 2 |  | | 1 | 1 | 2 | |  | | | |  | | | | | | | | 1 | | | | 1 | |  | | | | | 2 | |  | | Stockley 2001; WHO 2013 |
| Identification, training, monitoring, and feedback for those who are responsible for implementation | | | 2 | 2 | | 2 | 1 | 4 | | 1 | | | |  | | | | | | | | 4 | | | | 1 | | 1 | | | | | 5 | |  | | Stockley 2001; ADA 2003; D Dixey 1999; Spencer 2013; WHO 2013 |
| Plans for communication between parties involved in adoption of the program | | | 3 | 1 | |  |  |  | |  | | | | 1 | | | | | | | | 1 | | | |  | |  | | | | | 1 | |  | | Besculides 2008 |
| Partnering with community organizations in implementation processes | | | 3 | 1 | |  |  |  | |  | | | | 1 | | | | | | | | 1 | | | |  | |  | | | | | 1 | |  | | Besculides 2008 |
| Partnering/consultations during the development of implementation strategies | | | 3 |  | | 1 |  | 1 | |  | | | |  | | | | | | | | 1 | | | |  | |  | | | | |  | | 1 | | Bellew 2008 |
| *Implementation consistency, and adaptation processes* | | |  |  | |  |  |  | |  | | | |  | | | | | | | |  | | | |  | |  | | | | |  | |  | |  |
| Implementation consistency and adaptations made during delivery | | | 1 | 2 | |  |  |  | | 1 | | | | 1 | | | | | | | | 2 | | | |  | |  | | | | | 2 | |  | | Rabin 2010; Brennan 2014 |
|  | | | 2 | 3 | |  |  | 3 | |  | | | |  | | | | | | | | 3 | | | |  | |  | | | | | 3 | |  | | NCI 2012; Netherlands Centre Youth Health 2013; IMPALA 2011 |
|  | | | 3 | 1 | |  |  | 1 | |  | | | |  | | | | | | | | 1 | | | |  | |  | | | | | 1 | |  | | Glasgow 2007 |
| Adherence to protocol/protocol fidelity | | | 1 | 4 | | 1 | - | 1 | | - | | | | 3 | | | | | | | |  | | | | 4 | | - | | | | | 2 | | 2 | | Kohl 2013; Ghisi 2014; Goode 2013; Vuillemin 2011 |
|  | | | 3 | 1 | |  |  | 1 | |  | | | |  | | | | | | | | 1 | | | |  | |  | | | | | 1 | |  | | Glasgow 2007 |
| Implementation strategies specified and disseminated | | | 2 | 2 | |  |  | 1 | | 1 | | | |  | | | | | | | | 1 | | | |  | | 1 | | | | | 2 | |  | | Simovska 2010; Dubois 2008 |
| Implementation process described and measured (e.g., time management, type of activities) | | | 2 | 2 | |  |  | 2 | |  | | | |  | | | | | | | | 1 | | | | 1 | |  | | | | | 2 | |  | | Netherlands Centre Youth Health 2013; WHO 2011 |
| Implementation quality assessment and quality management | | | 2 | 1 | | 1 |  | 2 | |  | | | |  | | | | | | | | 1 | | | |  | | 1 | | | | | 2 | |  | | Netherlands Centre Youth Health 2013; IMPALA 2011 |
|  | | | 3 | 1 | |  |  | 1 | |  | | | |  | | | | | | | | 1 | | | |  | |  | | | | | 1 | |  | | King 2011 |
| Plans for implementation consistency | | | 3 | 1 | |  |  |  | |  | | | | 1 | | | | | | | | 1 | | | |  | |  | | | | | 1 | |  | | Besculides 2008 |
| Time frame for implementation | | | 3 |  | | 1 |  | 1 | |  | | | |  | | | | | | | |  | | | |  | | 1 | | | | | 1 | |  | | Christiansen 2014 |
| Delegation of responsibilities in implementation process | | | 3 |  | | 1 |  | 1 | |  | | | |  | | | | | | | |  | | | |  | | 1 | | | | | 1 | |  | | Christiansen 2014 |
| Roles of agencies/stakeholders in the implementation process specified | | | 3 |  | | 1 |  | 1 | |  | | | |  | | | | | | | | 1 | | | |  | |  | | | | |  | | 1 | | Bellew 2008 |
| Barriers to implementation (e.g. access) | | | 3 |  | | 1 |  | 1 | |  | | | |  | | | | | | | | 1 | | | |  | |  | | | | | 1 | |  | | Kahn 2002 |
| Implementation auditing and monitoring criteria established | | | 1 | 1 | |  |  |  | | 1 | | | |  | | | | | | | | 1 | | | |  | |  | | | | | 1 | |  | | Richardson 2013 |
| Research into the realization of program implementation | | | 2 | 1 | |  |  | 1 | |  | | | |  | | | | | | | | 1 | | | |  | |  | | | | | 1 | |  | | Netherlands Centre Youth Health 2013 |
| Implementation across different types of infrastructures | | | 2 |  | | 1 |  | 1 | |  | | | |  | | | | | | | |  | | | |  | | 1 | | | | | 1 | |  | | IMPALA 2011 |
| *Transferability* | | |  |  | |  |  |  | |  | | | |  | | | | | | | |  | | | |  | |  | | | | |  | |  | |  |
| Transferability (can be transferred to other populations/communities, other settings, other cultures) | | | 1 | 1 | | 1 |  | 1 | | 1 | | | |  | | | | | | | | 2 | | | |  | |  | | | | | 2 | |  | | Flynn 2006; Hoehner 2013 |
|  |  |  | 2 | 4 | |  |  | 3 | | 1 | | | |  | | | | | | | | 3 | | | | 1 | |  | | | | | 4 | |  | | Netherlands Centre Youth Health 2013; Khan 2009; Dixey 1999; Spencer 2013 |
| Adaptation procedures for transfer | | | 2 | 2 | |  |  | 2 | |  | | | |  | | | | | | | | 2 | | | |  | |  | | | | | 2 | |  | | Dubois 2008; Netherlands Centre Youth Health 2013 |
| Contexts of transfer and transfer boundaries | | | 2 | 3 | |  |  | 3 | |  | | | |  | | | | | | | | 3 | | | |  | | - | | | | | 3 | |  | | Dubois 2008; Netherlands Centre Youth Health 2013; Spencer 2013 |
| Active components for successful transfer | | | 2 | 1 | |  |  | 1 | |  | | | |  | | | | | | | | 1 | | | |  | |  | | | | | 1 | |  | | Spencer 2013 |
| Political/social/economic conditions for transfer | | | 2 | 1 | |  |  | 1 | |  | | | |  | | | | | | | | 1 | | | |  | |  | | | | | 1 | |  | | Spencer 2013 |
| Transferability based on a common frame but flexible protocols (e.g., allowing for cross-country adaptations) | | | 3 | 2 | |  |  | 1 | | 1 | | | |  | | | | | | | | 2 | | | |  | |  | | | | | 2 | |  | | Summerbell 2012; Glasgow 2007 |

Note: Document type: 1 = systematic review, 2 = stakeholders’ document, 3 = position review paper; for columns ‘Population’, ‘Type of behavior”, ‘Policy/intervention’ and ‘Number of levels’, the numbers in columns indicate the number of documents addressing respective type of population, type of behavior, policy or intervention and single or multiple levels addressed in policy/intervention.
